# Identifying Mucormycosis Severity in Indian COVID-19 Patients: A Nano-Based Diagnosis and the Necessity for Critical Therapeutic Intervention

**DOI:** 10.3390/antibiotics10111308

**Published:** 2021-10-27

**Authors:** Syed Mohammed Basheeruddin Asdaq, Arya Rajan, Aswin Damodaran, Shivali R. Kamath, Krishnanjana S. Nair, Subin Mary Zachariah, Ram Kumar Sahu, Santosh Fattepur, Nagaraja Sreeharsha, Anroop Nair, Shery Jacob, Hussain A. Albahrani, Eman H. Alkhaldi, Yahya Mohzari, Ahmed A. Alrashed, Mohd. Imran

**Affiliations:** 1 Department of Pharmacy Practice, College of Pharmacy, AlMaarefa University, Riyadh 13713, Saudi Arabia; 2 Amrita School of Pharmacy, AIMS Health Science Campus, Amrita Vishwa Vidyapeetham, Kochi 682041, India; arya01234rajan@gmail.com (A.R.); daswin25@gmail.com (A.D.); kamathshivali@gmail.com (S.R.K.); krishnanjana99@gmail.com (K.S.N.); 3 Department of Pharmaceutical Science, Faculty of Pharmacy, Universitas Airlangga, Surabaya 60115, Indonesia; ramkumar.sahu@aus.ac.in; 4 Department of Pharmaceutical Science, Assam University (A Central University), Silchar 788011, India; 5 School of Pharmacy, Management and Science University, Shah Alam 40100, Malaysia; dr_santosh@msu.edu.my; 6 Department of Pharmaceutical Sciences, College of Clinical Pharmacy, King Faisal University, Al-Hofuf 31982, Saudi Arabia; sharsha@kfu.edu.sa (N.S.); anair@kfu.edu.sa (A.N.); 7 Department of Pharmaceutics, Vidya Siri College of Pharmacy, Bangalore 560035, India; 8 Department of Pharmaceutical Sciences, College of Pharmacy, Gulf Medical University, Ajman 4184, United Arab Emirates; dr.sheryjacob@gmu.ac.ae; 9 Pharmacy Department, Dammam Medical Complex, Dammam 32245, Saudi Arabia; Huaalbahrani@gmail.com; 10 Pharmaceutical Care Services, King Saud Medical City, Riyadh 12746, Saudi Arabia; e.alkhaldi@ksmc.med.sa; 11 Clinical Pharmacy Department, King Saud Medical City, Riyadh 12746, Saudi Arabia; N3536999@kfshrc.edu.sa; 12 Pharmaceutical Services Administration, Inpatient Department, Main Hospital, King Fahad Medical City, Riyadh 11564, Saudi Arabia; aahalrashed@kfmc.med.sa; 13 Department of Pharmaceutical Chemistry, Faculty of Pharmacy, Northern Border University, Rafha 91911, Saudi Arabia; mohammad.baks@nbu.edu.sa

**Keywords:** COVID-19, mucormycosis, nanotechnology, antifungal, diabetes, diagnosis

## Abstract

The COVID-19 infection caused by the new SARS-CoV-2 virus has been linked to a broad spectrum of symptoms, from a mild cough to life-threatening pneumonia. As we learn more about this unusual COVID-19 epidemic, new issues are emerging and being reported daily. Mucormycosis, also known as zygomycosis or phycomycosis, causes severe fungal illness to individuals with a weakened immune system. It is a devastating fungal infection, and the most frequent kind is the rhino cerebral type. As a devastating second wave of COVID-19 sweeps India, doctors report several instances involving a strange illness—sometimes known as the “black fungus”—among returning and recovered COVID-19 patients. This paper analyzes the existing statistical data to address the severity of prevalence and further notes the nano-based diagnostic parameters, clinical presentations, its connection with other conditions like diabetes, hypertension, and GI disorders, and the importance of anti-fungal therapy in treating the same. Anti-fungal therapies, as well as surgical interventions, are currently used for the treatment of the disease. Proper and timely diagnosis is necessary, along with the reduction in the spread of COVID-19. From the review, it was found that timely pharmacologic interventions and early diagnosis by using a nano-based diagnostic kit can help control the disease. Additionally, this paper provides novel information about the nanotechnology approaches such as fungal detection biosensors, nucleic acids-based testing, point-of-care tests, and galactomannans detection, in the diagnosis of mucormycosis, and thereby reinforces the need for further research on the topic.

## 1. Introduction

COVID-19, also known as severe acute respiratory syndrome, is caused by the coronavirus. Disease statistics show immense variations in terms of mutation and mortality rates. Since the emergence of the disease, several studies have been conducted in terms of pathophysiology, diagnosis, treatment, prognosis, and complications [1], many of which are unexpected and unreliable. Other than the most common symptoms such as shortness of breath, fever, diarrhea, nausea, throat pain, and dizziness observed, these clinical presentations varied both geographically and in epidemiological analysis. High-grade fever and heart injury were also evident. In co-morbid patients, symptom severity was greater, and complications worsened the condition [2]. According to the statistical analysis, variations and mutations have emerged due to differences in environmental conditions, climatic change, and locality. Co-morbid patients with low immunity, such as cancer patients and patients who are on immunosuppressants, due to their very low immunity, cannot withstand severe infections and the pandemic can change the entire scenario of these patients.

Many make the diagnosis at early periods, which reduces the risk of complications. Nevertheless, many young adults aged 30–50 years, who may be co-morbid, lack immune mechanisms and even infection is bizarre for them. On such occasions, people living in the tropics were presented with a varied fungal infection, and symptoms were frightening, such as black colored hands, heart, and even brain tissue. Initial cases were sporadic, and infection was unnoticed. Later times, through invasive diagnosis and collection of culture samples, fungal sinusitis was commonly called mucormycosis. Its cause was unknown, yet on examination of the middle ear cavity and nasal septum, hyphal spores were found in the sinus tissue [3,4]. Many were panicked due to its unpredictable symptoms such as discoloration of the eyes, ear cavity, and nasal cavity. The disease was later presented as Rhino cerebral mucormycosis. In severe cases, it can lead to nasal septum obstruction, edema, ptosis, and even ophthalmoplegia. Black eschar was found in the nasal cavity in patients rushed to the hospital or previously hospitalized in ICU [5,6]. Mycotic blood vessel infiltration, infarction, thrombosis, hemorrhage, and chest pain are other histopathological characteristics [7].

The condition can advance significantly without early discovery and treatment, with 50–80% fatality rates reported from intra-orbital and cerebral complications [8].

Infection typically spreads, resulting in mortality. Many of those patients were steroid-dependent or had any history of steroids, and many had other medical conditions such as diabetes, hypertension, cancer, and asthma [9]. Recently, the number of cases of mucormycosis, with sinus inflammation as the symptom, has increased, with more instances being recognized and diagnosed more frequently.

Secondary infections can be caused by a dynamic interaction of factors such as asthma, previous respiratory pathology, immunosuppressive medication, or any other nosocomial infection sources [10]. Due to lower levels of Cluster of differentiation 4+T cells and 8+T cell counts, they are more susceptible to fungal co-infections [11]. Mechanical breathing was essential, and almost all severe patients required it due to the risk of secondary infections and those acquired in the hospital. Food and the surrounding environment also had a role in elevating the disease complications [12]. The COVID-19 pandemic has dramatically increased steroid usage, and supplements such as zinc and iron tablets have increased this fungal disease’s severity. Hence, differential diagnosis gives the source of the infection more accurately, and scientists are startled by the fact that meager changes in human health can cost lives for several [13].

Recently, patients affected and recovering from COVID-19 worldwide have been suffering from fungal infection. Many cases have been reported in countries like India, where it has affected approximately 72,000 people [14]. India’s Health Ministry has ordered the state governments to proclaim it a acquaint diseases under the Epidemic Act. To elaborate, healthcare professionals should track every case and take the necessary steps. Maharashtra has more than 2000 cases, Gujarat has about 1200 cases, and the rest of the states are also affected by the infection [15]. Karnataka alone has recorded about 700 cases as of 17 May 2021 [16].

## 2. Clinical Presentations

Mucormycosis, also known as the “black fungus”, was found to be high among COVID patients.

Some of the standard clinical presentations include:

- Headaches that are unbearable: When a patient inhales the fungal molds, which target the nasal canals and nerves, the fungal infection can be very dangerous. As a result, a person may have symptoms such as chronic discomfort and headaches.
- Sinusitis—nasal blockage or congestion, nasal discharge (blackish/bloody).
- Local pain on the cheekbone, one-sided facial pain, numbness or swelling; these might be critical indicators of infection right now. Aside from swelling, the fungal infection may impact skin health, causing numerous lesions and necrosis-like signs.
- Blackish discoloration over the bridge of the nose/palate. The infection’s most noticeable symptom is facial deformation. In the most severe cases, the infection can cause black patches to appear around the eyes and nose. In certain circumstances, random fungal infection development might result in a person losing teeth or jaws.
- Tooth decay, jaw involvement.
- Black spots in front of eyes and pain with blackish pupil.
- Experts warn that changes in the eyes or visual distortion might be indicators of the illness progressing. Vision might be affected as the black fungus develops and spreads. Some people may also get bloodshot eyes, have cloudy or impaired vision, or feel swelling in one eye.
- Thrombosis, necrosis, skin lesion [17].
- Doctors warn that important symptoms such as delirium, memory loss, neurological damage, and changed mental state might signal that a patient needs medical treatment.
- Pain in chest, pleural effusion, problem in respiratory symptoms.
- Black fungus is most evident among high-risk patients as per the clinical findings, people on continuous steroid use or immunomodulators and diabetic patients and those who are on prolonged ICU stay post-transplant/malignancy.

## 3. Pathological Evidences

**Figure 1:**
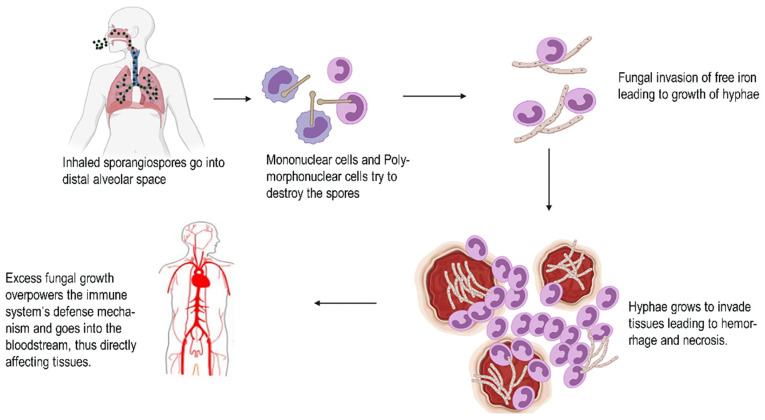
Pathological evidence of black fungus.

In ICU patients infected with COVID-19, oxygen delivery is crucial; oxygen must be highly sterilized and highly purified, such that before administration, oxygen must undergo humidification. When oxygen is provided without being humidified, it dries out the mucous membrane and damages the lungs’ inner lining. The phlegm or discharge will become quite viscous, making it difficult to expel. Spores called “Mucorales” enter through the nasal septum, and untimely patients can get black sputum, inflammation, and stuffy nose, and other symptoms of black fungus infection. Direct entry through the nose is followed by entry into the blood vessels when the immunity is low, and spreads to other parts of the body [18] (Figure 1).

Continuous steroid use is dangerous and harmful; steroids usually reduce inflammation in the body. In addition, the administration of steroids or their early use can reduce body immunity, mainly decreasing phagocytes [19]. A decrease in phagocytes increases the Mucorales spread in the blood vessels by which infection can spread rapidly.

In diabetic patients and non-diabetic patients, mucormycosis increases the blood sugar, as sugars help in the storage of spores of the fungi; hence, it rapidly increases the body’s blood sugar levels as soon as it enters the body, anchors on the sugar molecules, utilizes simple sugars and spreads to produce more complex carbohydrates [20].

The fungi that cause mucormycosis are *Rhizopus Oryzae* and release the sporangiospores *R. oryzae*. These are predominantly present in the surrounding environment, contaminated food, contaminant oxygen cylinders, or from contaminated soil.

When immunocompromised patients inhale the air containing spores or swallow, when there are spores in food or spores contaminated spores, the spores directly enter the lungs. Spores can be 1000–2000 per cubic meter in the outdoors [21]. When there is an increased amount of sugar in the blood, it acts as a carrier for these sporangiospores. Sporangiospores then spread through the blood into broader areas in the body through blood vessels. It mainly affects the eyes, nose, lungs, and brain.

Fungi belong to mucoralean that includes the genus of mucor and rhizopus. Spores released from the sporangiospores are dark-hued structures, microscopic, and spherical. Spores dispersed into the air when they stick on moisture-like surfaces. They germinate and produce a thread-like structure called mycelium in which threads are branched and attached to the sugars. On attachment, another mycelium grows through germination [22]. Fungi utilize simple carbohydrates and produce more complex sugars such as cellulose on germination, spreading to vital organs and tissues and producing blackish discoloration on the site.

When the body’s immune system is drastically reduced during cancer, AIDS, or organ transplantation for organ rejection, the fungal spores that produce hyphae get hooked on blood sugars. Phagocytes generally engulf the spores and clean the blood in normal people. On the fungal attack, it reduces the phagocytes immensely in low-immunity patients. Germination of mucoraceae spores increases, and infects the tissues, mainly the lungs in the alveolar wall, sinuses, in the pupil (eye), and in the brain [23]. This leads to the formation of black crusts, swelling in and around the nasal passage, and the eyes becoming peculiar.

Dark colored spores accumulate on the skin; spores also accumulate in the brain and blood vessels. Usually, an attack on the eye is treated with removal of the eye to prevent its spread to the brain.

Pulmonary alveolar macrophages are highly exposed to the fungi and cannot stop germination. As a result, fungal spores that disseminate into the nasal cavity or the sinuses lead to rhino cerebral disease [24]. If it enters the intestines, it leads to disease called GI and if it is through the cuts in the skin or wounds, it can lead to cutaneous disease.

On invasive diagnosis, fungal spores spread and are found within the nasal sinuses at an obtuse angle followed by rhinitis, stuffy nose, and discoloration of the nasal septum. It is then branched into the lungs. All T cells, CD4 cells, and CD8 cells are tremendously reduced, followed by lowered innate immunity, which further spreads to the lungs.

The spread of the infection is rapid, and within three days, it infects the eye and jaw bone. The orbit spreads to the brain through the esophagus into the GIT, and along the trachea, it infects the lungs. CT of the brain shows fungal lesions behind the forehead, mostly between occipital and temporal lobes, yet findings are unknown. Invasive endoscopy of the intestines also does not show any clinical findings [25]. When immunity drops, fungal infection rises and is primarily seen in tropical regions where spores are immensely dispersed in the air and when people are vulnerable to the disease.

## 4. Statistical Data of Mucormycosis Cases and Steps Taken

A worldwide rise in casualties with mucormycosis has been observed, but most cases were found mainly in India and China. Diabetes and the use of steroids are some of the most prominent risk factors [26]. However, another study showed that most cases had their origin from Europe and Asia when about 851 patients were studied from 2000 to 2017. The detailed percentage statistics are as follows: Europe (34%), Asia (31%), North or South America (28%), Africa (3%).

The count of patients hospitalized with mucormycosis was estimated to be about 0.12 per 10,000 discharges in 2005–2014, as evidence of a retrospective case study conducted in USA [16]. A similar study from Spain reported a prevalence of 3.3 cases among 100,000 admissions until 2015 [27,28]. Switzerland faced a hike in mucormycosis cases to 6.3 cases per 100,000 admissions due to increased Voriconazole and Caspofungin as a prophylactic agent [18].

From 2013 to 2015, India reported 89 cases in a single hospital, pointing fingers at a massive prevalence of the disease in the country. Reports from the Leading International Fungal Education (LIFE) are indicative evidence; the WHO estimated the burden of mucormycosis infection and found that the annual prevalence can be around 10,000 except in India. When the Indian cases were added, the number rose to 91,000 cases per year [29].

Even now, the country is not free from infection. As per reports from Delhi, about 760 cases are still being treated in hospitals [30], while Tamil Nadu has reported 3300 cases and 122 deaths as reported on 3 July 2021.

All over the country, special wards were arranged at medical college hospitals and district headquarters hospitals. In the case of private hospitals, the drugs needed to treat them will be directly given to them by the central and respective state governments [31].

The Indian Council of Medical Research recently released its guidelines for the early detection of infection. These guidelines advise that patients who recovered from COVID-19 should thoroughly check for symptoms and seek treatment at the slightest indication of any clinical presentation. The treatment opts for a multidisciplinary approach with the help of ophthalmic surgeons, ENT specialists, general surgeons, neurosurgeons, dental surgeons, etc. Currently, the drug of choice is Amphotericin-B and Posaconazole, and Health Minister Dr. Harsh Vardhan asked the patients not to self-medicate with steroids as this can increase the detrimental effects of the infection [32]. The ICMR guidelines cite the management of disease through controlling diabetes, reducing steroids and immunomodulatory drugs, etc. Emergency medical treatment include installing an outlying inserted central catheter, maintained adequate hydration, infusion of normal intravenous saline before administering Amphotericin B, and anti-fungal therapy for at least 4–6 weeks.

### Mucormycosis Outbreaks

Mucorales and Entomophthorales are the two orders that make up the *Zygomycetes* class. These two groups produce infections that are very distinct from one another. Mucormycosis is a vascular infection caused by Mucorales fungi (*Rhizopus*, *Mucor*, *Rhizomucor*, *Absidia*, *Apophysomyces*, *Cunninghamella*, and *Saksenaea*). *Mucormycosis* can damage the rhino-orbito cerebral region and the lungs, skin, and gastrointestinal tract.

The first instance of mucormycosis was diagnosed as *Mycosis Mucorina* by the German physician Paltauf in 1885. Mucormycosis agents must scavenge enough iron from the host to mature, avoid the party giver phagocytic defenses, and get access to the vasculature to disperse to cause sickness [33]. In a healthy person, primary defense mechanisms against mucormycosis include specialized iron-binding proteins that sequester iron in the blood, phagocytes such as circulating neutrophils and tissue macrophages, and endothelial cells that modulate vascular tone and permeability. When these systems function together, they prevent tissue infection and endovascular invasion. Invulnerable hosts, standard defense systems are impaired [34,35,36,37,38,39].

**Figure 2:**
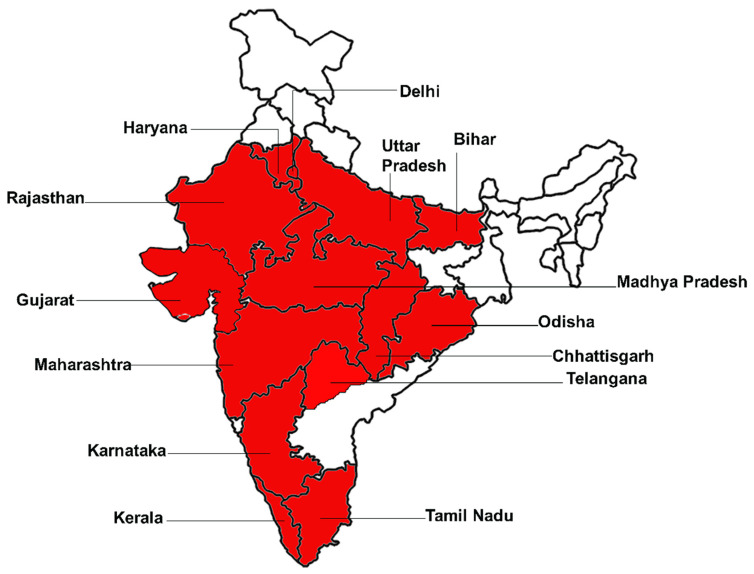
States with confirmed cases of black fungus after COVID infection in India. Red color indicates states in India with reported cases.

Figure 2 represents the states with confirmed cases of black fungus after COVID infection in India. Red color indicates states in India with reported cases. The number of instances of black fungus in Andhra Pradesh has risen to 4597, according to the state’s health department. In the last week, the state has seen 151 instances of mucormycosis, with 14 deaths reported. According to the health department, 225 people were able to recuperate throughout the same time frame. A total of 428 individuals have died as a result of the infection so far, with 3492 people recovering. During the past week, the number of active cases in Andhra Pradesh fell by 88. The total number of active cases is now 677. Anantapuramu District has recorded 32 instances, Guntur has reported 21, Kadapa has reported 16, and East Godavari has reported 13. For the third week in the running, Vizianagaram district saw three new cases, while West Godavari had none. In the meantime, Chittoor had six deaths in a week, East Godavari had four, Visakhapatnam had two, and Kurnool and Anantapuramu each had one. There were no deaths as a result of the virus in the remaining eight districts [40].

Mucormycosis was on the rise in the 1980s and 1990s, especially among immunocompromised patients [38]. The mucor mold, which may be found in soil, seeds, manure, and rotting fruits and vegetables, causes mucormycosis. It affects the sinuses, brain, and lungs, and people with diabetes and those with weakened immune systems, such as cancer patients or HIV/AIDS patients, are at risk. Doctors suspect that the administration of steroids, a life-saving treatment for COVID-19 patients who are extremely unwell, is causing mucormycosis, which has a 50% fatality risk [41]. By lowering inflammation in the lungs, steroids can help minimize some of the harm that can occur when the immune system overdrives to fight the corona virus. They weaken immunity and elevate blood sugar levels in both diabetic and non-diabetic COVID-19 patients. This loss of immunity is assumed to be the cause of mucormycosis outbreaks.

The mucormycosis (black fungus) epidemic has surged in India, not only by the incorrect use of steroids among diabetic COVID patients but also by the overuse of antibiotics, zinc supplements and iron pills. As the go-to immune boosters in COVID patients, zinc supplements have been given since the rise of the pandemic. As a result, zinc supplements have emerged as one of the highest, best-selling drugs in the country in the year 2020. The mucormycosis outbreak, a rare but deadly infection, has risen rapidly, with more than 8000 cases until May 2021. Recent studies revealed that growth was difficult without zinc. According to the studies from the data collected, molds of mucormycosis called mucormycetes can increase with zinc and act as a growth factor for the fungus. Therefore, with increased research studies, zinc and iron pills have been one of the major etiologic factors for mucormycosis.

## 5. COVID-19 and Fungal Co-Infections: A Diagnostic Perspective

More scientists are becoming aware of fungal infections as the coronavirus outbreak progresses. According to the French Council for Public Health, fungal infections should be examined regularly in coronavirus patients [42]. Many academicians who have a lot of experience with COVID-19 therapy have advised doctors to pay great attention to their patients’ fungal infections, especially if they are sick or immunocompromised [43]. The early stages of the disease may manifest as abnormal chest imaging or extrapulmonary fungal infections. Tiny, focal lesions may often be surgically excised before they progress to include vital structures or disseminate, so early diagnosis is essential. Rapid diagnosis, reversal of the baseline confounding factors, effective surgical removal of contaminated tissue, and proper use of anti-fungals in management are also essential factors in eradicating mucormycosis. As a result, seriously ill patients must be monitored for fungal infections, which include, for suspected patients, the following tests that should be performed:

1. Direct microscopy and culture are used to examine the etiology.
2. Histopathology.
3. Serology: Similarly, tracheal suction (TA) and bronchoalveolar lavage liquid (BALF) tests for culture and biomarker testing should be done in well-protected environments due to the risk of airborne dissemination and contamination of healthcare specialists. On the other hand, antigen and galactomannan (GM), and counteracting agent, BDG [38], discovery by serum are also necessary for suspected individuals.
4. Pathogens can be identified using real-time polymerase chain reaction (PCR) techniques and, if applicable, molecular recognition. PCR methods and atomic acknowledgement on the off chance that appropriate [43] can be perceived.

Anti-fungal susceptibility scanning (AST) may be used to evaluate susceptible anti-fungal drugs after the pathogen has been identified. If the AST cannot be performed, it can be handled on a case-by-case basis. *Aspergillus* and *Candida* are the most common fungi that cause fungal co-infections in COVID-19 severe patients. Mucor and Cryptococcus, two less common opportunistic pathogenic fungi that cause lung infections, must also be recognized.

### 5.1. Invasive Mucormycosis (IM)

COVID-19 individuals with diabetes, trauma, GC use, HM, allo-HSCT, chronic neutropenia, and SOT are more likely to develop mucormycosis [44]. The nearness of non-septate or pauci-septate Mucorales hyphae with a variable width of 6–16 lm in clinical examples such as sputum, BALF, and skin injuries, as well as the nearness of fluorescent brighteners in clinical examples such as sputum, BALF, and skin injuries, is broadly suspected of mucormycosis. To confirm the diagnosis, tissue sections stained with Hematoxylin-Eosin (HE), PAS, or GMS should show non-pigmented hyphae demonstrating tissue invasion [45]. For genus and species identification, specimen culture, as well as AST, is strongly suggested. It is recommended that it be grown in two independent cultures at 300 °C and 370 °C, where a cottony white or greyish black colony is usually apparent, followed by fungal morphological identification or DNA sequencing utilizing bar code genes such as 28S, 18S ITS, or rDNA, as well as other methods. Due to the fact that MALDI-TOF identification relies on in-house databases, which many laboratories do not have, it receives relatively little support [46]. It is also possible to detect fungal DNA in blood and other bodily fluids, as well as paraffin-embedded tissue, but only with modest sensitivity due to a lack of homogeneity in the field.

### 5.2. Nanotechnology Based Mucormycosis Diagnosis

The range of medical tools, information, and therapies available to clinicians is already expanding because of nanotechnology. Nanomedicine, or the application of nanotechnology to medicine, makes use of the natural scale of biological activities to provide precise disease prevention, diagnostic, and treatment options [47]. In commercial uses, gold nanoparticles have been modified as probes for the detection of targeted nucleic acid sequences, and gold nanoparticles are also being investigated therapeutically as potential cancer and other sickness treatments [48]. Recent medicinal breakthroughs have the potential to enhance mucormycosis outcomes. The cornerstone of primary therapy for mucormycosis has evolved into Lipid Formulations of Amphotericin B (LFAB).

#### 5.2.1. Approaches to Mucormycosis Diagnosis Using Nanotechnology

##### Fungal Detection Biosensors

Current and future advancements in biosensor technology, which include a number of procedures now not formerly utilized in medical mycology, are predicted to noticeably resource fungal diagnostic research. Biosensor technology are anticipated to play an extra position within the diagnosed and monitored of all infectious ailments, but they have probable be mainly useful in the early detection of fungal infections. Additionally, biosensors allow non-stop monitoring of findings, which would possibly assist in evaluating an affected person’s reaction to therapy.

Analytical devices might be able to convert chemical, bodily, or biological facts into useable analytical indicators. According to International Biosciences records, it is done via the usage of an organic popularity detail (IUPAC). Sensors and biosensors are often used and function on the basis of generation of an electrical signal. Biosensor technology is a popular problem in research for the time being, with contributions from physicists, analytical chemists, biophysicists, and biologists [49]. The accompanying sections cover basic principles underlying the construction and operation of biosensors. We exhibit how those techniques at the moment are being utilized and talk capability potentialities for developing subsequent-era fungal diagnostic gear.

Most recently, scientists developed biosensors which are able to detect virulent genes specific to Aspergillosis fumigatus, which is the causative agent for invasive aspergillosis. This overcomes the disadvantages of the existing detection methods as they are complex and time consuming. The advantageous part is that scientists from the Indian Institute of Technology, Guwahati, and Centre for Cellular and Molecular Biology, Hyderabad, India, used chitosan-stabilized gold nanoparticles, which acts as a chemical linker and glip gene, a biomarker which is specific to the fungi and thus helps in the detection. The rationale is that when it is placed in a mixture containing a variety of gene sequences, the sensor selectively binds to the glip gene, thereby confirming the presence of the organism [50].

Researches also prescribe about a biosensor designed for the detection of Saccharomyces cerevisiae, owing to its robustness, genetic feasibility, and the safety profile of the system. They developed a single component biosensor which takes into account the fungal mating GPCRs so as to detect the presence of specific peptide components.

##### Working Principle of Biosensors

In general, a biosensor contains the following parts:

Bio-element;
Transducer;
Reference;
Amplifier;
Processor;
Display.

The recognition system and the transducer help produce a quantifiable signal on the basis of which detection occurs. The recognition system helps convert information from the bio-chemical product, basically referred to as an analyte, into a chemical signal. The transducer has the job of transferring the electric signal from the output region of the recognition system to that of the electrical domain for the result. The results are based on the binding affinities of the analyte. Antibodies are said to have high binding affinity and therefore give much better results. The transducer converts the chemical concentrations of the analyte into electrical signals, which are quantified, and thus the concentration is measured [51].

With the approach of targeting the nucleic acid sequences, fungal detection biosensors are able to detect the fungal infections at an early stage. The specificity, sensitivity, and accuracy also add to this advantage [52].

##### Diagnostics Based on Nucleic Acids

In-house Polymerase Chain Reaction (PCR) assays for the diagnosis of fungal infections have been developed and applied in a range of situations. Conventional PCR, real-time PCR (RT-PCR), nested PCR, PCR-ELISA, PCR utilizing the ITS and rDNA sections, multiplex PCR, and direct DNA sequencing are some of the molecular tests available [53]. This range of methods clearly offers advantages in terms of diagnostic specificity, since primers may be designed to detect specific illnesses; nevertheless, there are concerns about sensitivity and repeatability, particularly with regard to the creation of false negative findings. Traditional PCRs are quick and can enhance sensitivity, but there are no FDA-approved methods, so findings may vary between labs. [54]. This reality is well recognized, even in seasoned molecular laboratories that routinely use PCR methodologies, and efforts have been made to standardize various aspects of testing in accordance with guidelines developed by the European Aspergillus PCR Initiative (EAPCRI)/ISHAM (International Society for Human and Animal Mycology) research group. Recently, the American Thoracic Society advised for the collection of blood or serum samples in immunocompromised individuals to confirm Aspergillus infection. A modified “nested PCR” technique has been devised to increase specificity and sensitivity [55]. This is achieved by exposing samples to two consecutive PCR reactions using two sets of primers, which allows for the identification of fungal DNA at concentrations as low as 1 fg with 100% specificity; however, this is dependent on the sample type and concentration, and is especially susceptible to contamination [41].

Endoscopic sinus surgery specimens were shown to be especially inappropriate for this method, owing to high levels of fungal contamination in the nasal cavity from the environment. One downside of organism-targeted PCR is that it requires the formation of a hypothesis regarding the putative etiological agent’s nature [53]. Another option is to do a preliminary diagnosis using pan-fungal DNA amplification and then use target-specific primers to confirm the presence of specific fungal species [56]. This approach is less sensitive than utilizing specialized PCR primers since pan-fungal primers are designed to discover conserved sequences found throughout the kingdom. There are many commercial PCR tests available for Aspergillus and Candida, as well as commercial testing for mucoraceous molds, and Pneumocystis [57]. As per the recent study, when applied to serum samples, all commercial Aspergillus PCR tests demonstrated comparable sensitivity and specificity; however, serum samples had considerably lower sensitivity than respiratory samples. The MycAssay Aspergillus^®^ (Myconostica, Cambridge, UK) and AsperGenius^®^ (PathoNostics B.V., Maastricht, The Netherlands) tests were suggested for routine PCR detection of Aspergillus spp. DNA in respiratory samples. AsperGenius^®^ and MycoGenie^®^ (Ademtech, Pessac, France) are two of the kits that have the extra benefit of identifying the resistance markers TR34/L98H and TR46/Y121F/T289A associated with environmental azole resistance in A. fumigatus isolates from patients.

##### Point-of-Care Tests (POCT)

The College of American Pathologists defines a factor-of-care test (POCT) as “testing accomplished close or at the site of a patient with the end result main to a likely amendment within the patient’s care”. POCTs are presently carried out with little gadget via persons that do not have laboratory understanding, together with physicians, nurses, nursing assistants, and every now and then even the sufferers themselves, and encompass exams which includes domestic pregnancy trying out and blood glucose tracking. POCT diagnostics have a whole lot of ability for the front-line interventions in ailment remedy, and they can be divided into sorts: those that need little gadget and are easy to carry out, making them perfect for use in regions without state-of-the-art laboratory equipment, and those that use state-of-the-art techniques but are short to execute and may be miniaturized and run immediately (LOC) [58]. Although such gadgets could be expensive to purchase, they might be beneficial in resource-confined situations. The World Health Organization emphasizes the significance of them being inexpensive, sensitive, unique, resilient, and consumer-friendly, with portability being a key function [59]. In the area of scientific mycology, lateral flow devices with the usage of immunochromatography strategies at the moment are displaying the most promise, and are even occasionally getting used as POCTs. As previously mentioned, a commercial lateral float device for the detection of cryptococcal capsular antigen (IMMY Immuno-Mycologics, Norman, OK, USA) that can be used on finger-prick bloods has redefined the prognostic value of cryptococcal meningitis in developing countries where cryptococcal illness is a leading cause of death among HIV-infected populations (IMMY Immuno-Mycologics, Norman, OK, USA). A commercially available lateral go-with-the-flow instrument for the detection of Aspergillus antigen has also been developed (OLM diagnostics, Newcastle-upon-tyne, UK, USA). Thornton and associates (2008) commenced this via growing a monoclonal antibody (mAb JF5) that aims at an Aspergillus species early germ tube specific glycoprotein (JF5).

##### Detection of Galactomannans

Galactomannan (GM) is a widely used and early marketed Aspergillus biomarker. GM is a 20-kilodalton polysaccharide found in Aspergillus, Penicillium, and a few other fungus species. An immunoenzymatic sandwich microplate test for galactofuranosyl-containing compounds utilizing a rat monoclonal antibody specific for Aspergillus (despite cross-reactions with other fungus groups such as Fusarium, Aspergillosis patients with severe mucocitis have been reported to contain galactomannan in their blood [60]. Because high amounts of GM are produced only in the latter phases of invasive aspergillosis, generally after angioinvasion, the test may not be sensitive enough to detect the disease early. Serum GM can be utilized to predict result and assess antifungal medication response if a higher cut-off index value is employed. For pulmonary aspergillosis, the galactomannan test on BAL fluid is more sensitive and specific. The galactomannan test has the highest sensitivity and specificity when combined with other assays [61]. Table 1 highlighted the uses, advantages, and disadvantages of various types of nanotechnology based diagnostic tools.

## 6. Anti-Fungal Therapy as Challenging Aid to COVID-19

Even with the immense difficulties of treating critically ill patients with a single ridiculous infection, such as COVID-19 (for which there are currently no effective drugs), treating highly infectious patients with two potentially fatal infections is much more complex. This situation exacerbates COVID-19 along with fungal infection [66]. This can also be due to severe reduction in the arsenal of compounds commonly found in anti-fungals, resulting in dangerous drug reactions, high toxicity, and significant and extreme side effects such as kidney or liver damage.

COVID-19 reports show that a severe viral infection can harm multiple organs (including the liver, kidneys, and heart). When numerous infections were present, those safety issues were even more troublesome [67]. Specifically, when COVID-19 patients are combined with fungal infections, particularly those induced by multi-drug-resistant streptococcus mutants, the condition would be much more difficult. Several evolving fungal diseases have been shown to have novel resistance patterns; these restrict even the usable anti-fungal medication that is effective in treatment, making it a massive loss to both the pharmaceutical industry and patient care therapy [68].

**Figure 3:**
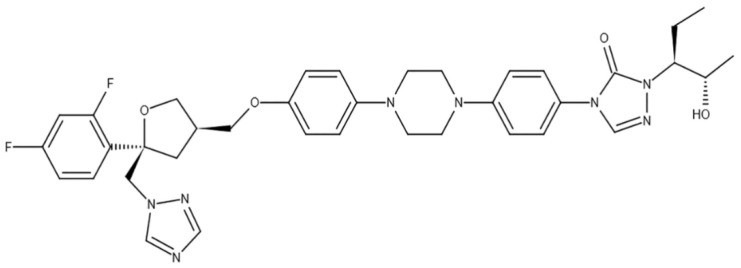
Chemical structure representing Posaconazole.

**Figure 4:**
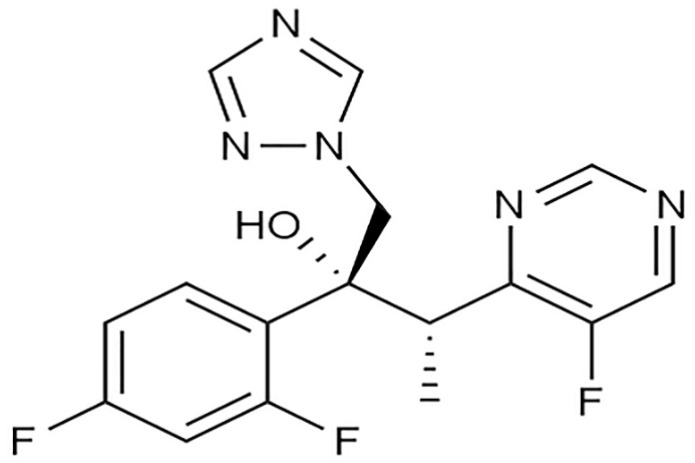
Chemical structure representing Voriconazole.

**Figure 5:**
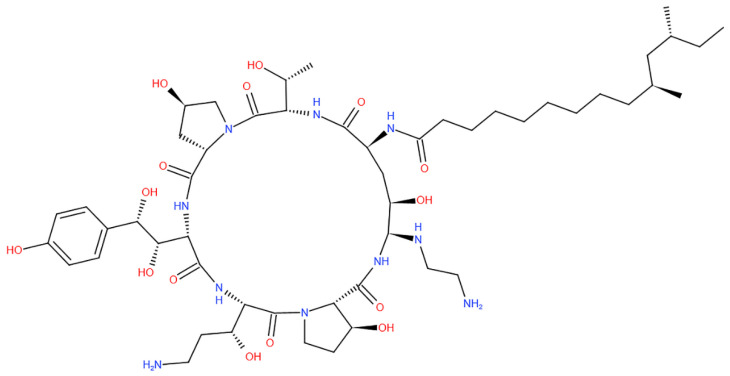
Chemical structure representing Caspofungin.

Extended therapeutic exposure to those novel triazoles (e.g., Posaconazole (Figure 3), Voriconazole, (Figure 4) and Isavuconazole) or echinocandins (e.g., Caspofungin, (Figure 5) Anidulafungin, and Micafungin) can lead which were once widely used can lead to resistance and therapy failure [67,68]. Commonly seen in people who have had extended exposure to anti-fungal drugs, several cases have already been documented. Possible drug-drug reactions during recovery are another factor that must be considered [69,70]. For COVID-19 treatment, a variety of medications are currently being studied or used empirically. Many trials have highlighted the medications Tocilizumab, interleukin (IL)-6 receptor antagonists, and glucocorticoids widely used to inhibit intense and dangerous inflammatory processes [71]. On the other hand, immune decline in the immune system has been shown to favor the emergence of opportunistic fungal infections.

**Figure 6:**
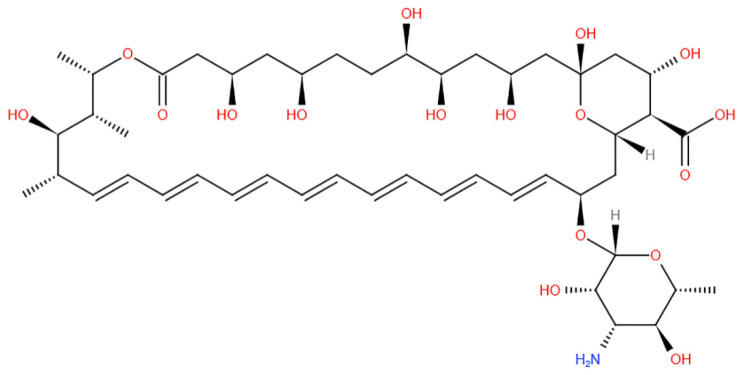
Chemical structure representing Amphotericin B.

Anti-fungals can be used to treat mucormycosis, but surgery may be needed in the end. According to physicians, controlling insulin, reducing steroid use, and discontinuing immunomodulating drugs are both critical [53]. To ensure proper systemic hydration, the procedure includes an IV infusion of daily saline (IV), supplemented by an infusion of Amphotericin B (Figure 6) and anti-fungal therapy infusion for at least 4–6 weeks [72]. Controlling hyperglycemia and regulating blood glucose levels during COVID-19 treatment, as well as in diabetics, were also highlighted by experts. Steroids should be used with caution; proper planning, dosage, and duration are essential. Unfortunately, the lack of available clinical trials makes it difficult for physicians to select from the existing anti-fungal agents for treating mucormycosis [73] (Table 2).

**Table 2:** Anti-fungal strategies for mucormycosis.

Therapy	Anti-Fungal Strategies	Pros and Cons	Mode of Action
Established therapies	Amphotericin B (AmB) deoxycholate	Toxicity	Depending on the concentration in body fluids and the susceptibility of the fungus, amphotericin B is either fungistatic or fungicidal. The medicine works by binding to sterols (ergosterol) in susceptible fungi’s cell membranes. This results in the formation of a transmembrane channel and a change in membrane permeability, which allows intracellular components to seep out. Amphotericin B and azoles act on ergosterol, the most abundant sterol in the fungus cytoplasmic membrane. Amphotericin B is a polyene that binds permanently to ergosterol, causing membrane rupture and cell death [74].
	Liposomal amphotericin B	Less toxic than AmB, most expensive polyene	Also acts by binding the sterols in fungal cell membrane. Binding cell membrane can cause alterations in cell permeability and also causes cell death [75].
	Amphotericin B lipid complex	Less toxic than AmB	Acts by binding to sterols in fungal cell membrane.
Investigational/adjunctive therapies	Itraconazole (Figure 7)	Superior toxicity profile	14-demythalase, a cytochrome P-450 enzyme required for the conversion of lanosterol to ergosterol, interacts with itraconazole. Because ergosterol is a necessary component of the fungal cell membrane, inhibiting its production causes increased cellular permeability, resulting in cellular contents leakage. Itraconazole can also decrease endogenous respiration, interact with membrane phospholipids, prevent yeasts from transforming into mycelial forms, prevent purine uptake, and affect triglycerides and phospholipid production [76].
	Posaconazole	More effective than itraconazole in animal models	Posaconazole is a triazole antifungal drug that works by blocking the cytochrome P-450 dependent enzymes sterols 14 demythalase in fungi by attaching to the heme co factor present on the enzymes [77]. This causes the synthesis of ergosterol, a critical component of the fungal cell membranes to be inhibited, as well as the accumulation of methylated sterol precursors. As a result, the fungal cell development is inhibited and eventually cell death occurs.
	Caspofungin	Very low toxicity, virtually no clinical data for mucormycosis	Caspofungin inhibits the synthesis of beta (1, 3) D-glucan, a key component of the cell wall of *Aspergillus* species and *Candida* species. In mammalian cells, beta (1-3)-D glucan is not found. The main target is beta (1,3)-glucan synthase [68].
	Iron chelation	Synergistic with ABLC in murine	Induces iron starvation and thus promotes a fungicidal effect.
	Hyperbaric oxygen	Benefit in combination with anti-fungals	Suppresses fungal growth in vitro, reduces tissue hypoxia and acidosis caused due to fungal invasion.
	Cytokine therapy	Nontoxic, Successful case reports	Cytokines play a key role in signaling molecules that modulate and control immunity, inflammation, and hematopoiesis. Cytokines are a diverse set of proteins, peptides, and glycoproteins released by the immune system of cells [78].

**Figure 7:**
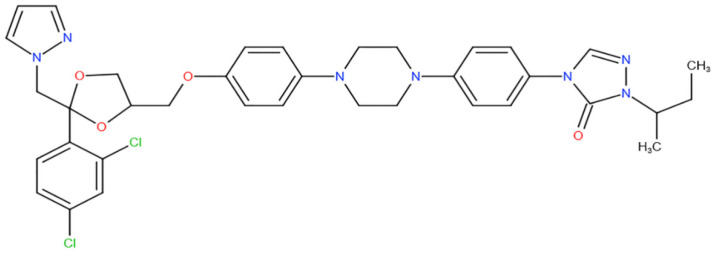
Chemical structure representing Itraconazole.

### Surgical Intervention and Management with Mucormycosis

Rhino cerebral disease with diabetes and maliciousness associated with neutropenia received spectrum antibiotics. Oral swelling and facial cells are progressed. There are black discharged noticed on face and cavity. Proptosis, ptosis, chemosis, and ophthalmological indicate re-orbital extension. Reduced level of consciousness in the mind shows the involvement of the brain [79].

Clinical data from 2000 to 2015 reveal that 35% of patients with lung illness had a micro biological diagnosis based on histopathologically consistent findings.

There was a meal preponderance with a mean age of 55 plus 15 years, according to the findings. Analysts of predisposing circumstances revealed the dominant incidence of millions. Sixty-six percent of patients are receiving immunosuppressive agents. The radiologic findings included pleural effusion and nodules. While all patients received medicinal treatment, 43% required further surgical surgery. An immunocompetent man who sustained a high-pressure water jet injury suffered from rhino cerebral mucormycosis. Excision of the right orbit, maxillary antrum, nasal cavity, sinus, and infratemporal fossa took place. The tissue was infarcted.

Surgical intervention is mandatory for a clinical presentation of necrosis followed by hemoptysis. Furthermore, the majority of pulmonary illness patients have hematologic malignancies as well as a history of neutropenia [80].

The vascular invasion that characterizes all types of cutaneous mucormycosis is shown in the developing black necrotic lesion. Patients with skin illness may have been exposed to contaminated medical equipment, such as bandages, in the past.

All four patients in an outbreak of cutaneous *Rhizopus oryzae* infection linked to sticky polyethylene tapes used to support peripheral venous catheters had hematological disorders, and the severity of the infection was proportional to the length of neutropenia. The infection has to be treated with systemic antifungal medication and surgical debridement in order to be resolved. The whole batch of tapes was recalled, and the outbreak was brought to a halt [81].

According to the case report, the patient was treated with extensive surgical debridement followed by an antifungal injection of amphotericin B lipid complex (Abelcet 5 mg/BW kg/day). The patient’s visual function was also compromised. The mucor-related effects were eradicated as a result of the severe surgical therapy that was followed up by a multidisciplinary team. The patient, however, died as a result of end-stage renal failure [82].

The literature has case reports of surgical therapy for primary cutaneous mucormycosis; however, the degree of debridement necessary for cure is unclear, and no standard treatment strategy has been proposed. There are currently no clinical recommendations available to help clinicians manage this condition surgically. This paper examines the research, presents two clinical cases, and offers clinical guidelines to help clinicians manage primary cutaneous mucormycosis surgically [83].

After an accident, a 21-year-old healthy man sustained polytrauma. He experienced a number of serious injuries, which are listed in Table 2. The patient had a Glasgow Coma Scale score of three on presentation, blood pressure of 96 mm hg, heart rate of 140 beats per minute, respiratory rate of 11 cycles per minute, and temperature of 35 degrees. Despite fluid resuscitation and blood product administration, the patient remained hypotensive and tachycardic. This necessitated rapid treatment of his abdominal and pelvic injuries in the operating room, where he was treated with surgical sponges for pelvis and abdomen discomfort [84].

The majority of verified mucormyscosis patients have open, severely infected sores in their bodies. The majority of mucormycosis infections have been found in military personnel who have died in combat. However, more recent occurrences have been discovered among civilians as a result of a motor vehicle accident. [85].

In addition, the patient had a series of operations and treatments, all of which result in pyrexia. Only when the patient’s pyrexia did not compromise the delivery of broad-spectrum antibiotics did more investigation become necessary. The key to managing and eradicating mucormycosis was surgical debridement; however, antifungal therapy is also commonly used in conjunction [86]. Amphotericin-B liposomal 3.0 mg/kg combined with vigorous surgical debridement, up to and including amputation, has been shown to be moderately successful in studies.

A 48-year-old woman infected with the same fungus and the fungal specimens was found on the cuff of a peritoneal catheter. The patient’s abdominal and genital problems improved temporarily after receiving intravenous amphotericin B. Clinical suspicion of intestinal perforation emerged 20 days after starting antifungal therapy, and an exploratory laparotomy was scheduled; however, the patient died during anesthesia induction. Deferoxamine was never given to the patient, and she had no factors that might make her susceptible to mucormycosis, such as diabetes or immunosuppression [87].

Duration of treatment, highly individualized, near normalization of radiograph, negative biopsy specimen and culture, and recovery from immunosuppression were all considered.

Removal of necrotic tissue increases penetration antifungals—lobectomy, pneumonectomy, or wedge resection. Necrotic tissue was completely removed, and salvage therapy or adjunctive therapy was initiated. For excellent disease management, it is advised to excise lung lesions if they are limited to a single lobe, excise cutaneous lesions completely, and resect any GI masses [88].

Even after recurrent antifungal therapy and adjunctive therapies, diagnosis for necrosis and antibiotic intervention worsens the infection of young patients. Therefore, radical surgical intervention or an aggressive surgical debridement is made mandatory to remove the necrosis tissue surrounding healthy tissue altogether. To treat an ascending and possibly spreading or uncontrolled infection, more drastic procedures such as hemipelvectomy or hemicorporectomy might be explored [89].

## 7. The Interplay between Black Fungus and SARS-COV-2

Many COVID-19 patients, including those who have recovered, have been infected with black fungus [90]. Due to the rising number of patients infected with COVID-19, this potentially lethal fungal illness is likely to spread. Fighting the coronavirus might weaken or damage people’s immune systems, increasing their risk of acquiring mucormycosis [45]. People with health issues (such as COVID-19) or those who take medications that reduce the immune system’s ability to combat infection are more likely to contract the virus. COVID-19 is the current epidemic, which causes the immune system to weaken. The patient’s body functions becomes weak and erratic opportunistic fungal infections if they already have a weakened immune system. Though there are numerous theories/explanations for the fast spread of mucormycosis in India, some of them seem more plausible than others. There are a few things to think about in order to come up with the best possible solution steps that must be taken in order to avoid such infections in the world [88].

Use of steroids to treat COVID-19 set the stage for mucormycosis: Steroid usage for an extended period decreases immunity and makes a person more vulnerable to fungal infection. As a result, excessive and incorrect usage of steroid therapy is unsuitable. The usage of steroid hormones is necessary using extreme caution and in accordance with the rules and guidelines [90]. Supplements of steroids are very well correlated with mucormycosis. As the dose of steroids given increases, it weakens the immune system and thus increases the chance of mucormycosis in patients with COVID-19. Patients with COVID-19 will have a weak immune system. Along with this, consumption of a high dose of steroids can increase the risk on the patients’ life and cause mucormycosis and various fungal infections. People using corticosteroids systemically have a higher risk of developing immune side effects than a drug taken topically or inhaled. Steroids can affect the immune cells and antagonize macrophage differentiation and suppress the production of macrophages, interleukin 1 and 6, TNF factors, etc. [91]. Regarding oral steroids, for example, patients with normal blood pressure should avoid it and it is also contraindicated with patients with the saturation of oxygen (SpO_2_). Self-medication is not recommended for people who have a mild to moderate infection and are on self-quarantine. Steroids have the ability to reduce inflammation of the lungs and help to stop the damage of the body due to a weakened immune system. The use of steroids can cause many side effects, mainly in immunocompromised patients and patients with diabetes. Steroids are basically life-saving in COVID patients for respiratory distress or in need of oxygen. Steroids can temporarily mask the patient’s symptoms, but within 4 to 5 days, the patient experiences high fever, respiratory involvement, and cough. If steroids are prescribed at the right dose and at the right duration, it will not be a major risk.

### 7.1. Underlying Conditions for Black Fungus in COVID-19

The black fungus is a deadly condition whose infection has led to a mortality rate between 46% and 96%. It is a rare infection that depends on the underlying conditions of the patients. Black fungus is a life-threatening disease across India especially during the second wave of COVID-19. The condition is caused by the Mucorales fungi group, which affects body parts, and is also known as mucormycosis. Once it affects the body parts, the condition spreads rapidly across the body and hence the rate of infection [92].

Research shows that COVID-19 patients especially in India are being affected by the novel disease at very high rates which have increased the death cases in the country. In India, the rate of infection of the disease for both the COVID-19 patients and the post-COVID-19 patients is a condition that has remained prevalent leading to massive deaths of people due to the high rate of infection, according to Daria et al. [93], of black fungus [94].

Out of the number of mucormycosis incidents, 86% of patients had a history of COVID-19, which made the medics conclude that the disease is highly associated with the COVID-19 pandemic. Sixty-two percent of the total cases recorded a history of diabetes, which also indicated that both COVID-19 and diabetic patients are at high risk of infection from the black fungus disease [95]. As a result, health experts have been projecting the actual prevalence of the disease to be higher than the reported cases.

The global data is way less than the actual prevalence of the disease according to research. For instance, it is recorded that the prevalence of the disease is 70 times higher than the COVID-19 cases across the world, which has accelerated the mortality rate [96]. Black fungus, according to research done by scientists, affects such parts of the body like the brain, the sinus, nerves, and the body tissues as well as the side of the face. Besides this, the victims might experience pain in the eyes, headache, blurred vision, and blindness. The conditions of the disease are very serious and, if untreated, they become fatal.

Once the patients have been infected, they suffer from loss of eyelids, blurred vision, and blindness. Therefore, physicians have had a hard time detecting the early signs of the disease for the victim [97]. In many cases, the patients hardly visit the health centers for treatment and the health experts detect the early signs of the disease leading to the high infection rate and hence death. Once the condition has been detected, it might force a particular physician to remove a part of the body like the eye to stop further spread [98].

Before the COVID-19 pandemic began in India, the country had never faced such a high rate of infection, which has challenged physicians and medical experts [99]. Most of them least anticipated such a disease as an opportunistic pathogen [100]. It affects the patients with low immunity because of the comorbidities and high level of steroid administration. Besides this, the patients are associated with oxygen therapy, poor hygiene especially in hospitals, organ transplants, and exposure to ventilation [101].

Mucormycosis can affect immunocompromised people, people with hematological malignancies, on chemotherapy, with diabetes, people who have undergone organ transplants, people with neutropenia, people living with HIV, and people using immunomodulation drugs. Diabetes mellitus can alter the normal response of the body to an infection in different ways. Hyperglycemic patients can stimulate fungal proliferation and decrease phagocytic efficiency, allowing organisms to live in an acid-rich environment. In patients with diabetic ketoacidosis, the organism Rhizopus oryzae produces an enzyme ketoreductase that enables them to use the ketone bodies. It is also said that patients with diabetic ketoacidosis can alter the ability of transferrin in binding to iron, which can lead to changes in defense mechanism and allow the growth of fungal organisms [102]. People with some hematologic malignancies are at high risk because of a condition called neutropenia. Conditions such as chemotherapy, people with some transplant history, or people living in HIV-infected areas are at high risk because of their weakened immune systems [103].

Mucormycosis infection has a high chance of getting an infection by use of steroids or by the weakened immune system, and it has a chance of causing more deaths than COVID-19. It is said that people with COVID-19 infection and with comorbidities like diabetes or people taking steroids have a high chance of contracting the fungal infection [104]. If we want to prevent mucormycosis, unwanted steroid use must be prevented. Using steroids like dexamethasone can repress the immune system. So, we should be careful regarding the use because it can suppress the immune system and can cause mucormycosis. Meanwhile, corticosteroids and TNF inhibitors are two classes of medications that show a high risk of causing fungal infections. Corticosteroids are medications that can treat various conditions like arthritis, asthma, inflammatory bowel disease, etc. If higher doses of steroids are given, it can affect the immune system of the patient [105].

A recent study done in India on black fungus patients indicated that 94% of the patients had diabetic conditions [106]. For COVID-10 patients, the exposure to humidity is an enabling factor during ventilation in the Intensive Care units. The opportunistic fungus is further accelerated by antibiotics and steroids, which increase the risk of infection [107]. Besides this, Indians have a tendency of alternating urine and cow dung for COVID-19 therapy as many people consume cow products assuming they are a COVID-19 cure. Such behavior can easily spread other diseases from animals [108]. For instance, cow dung is an opportunistic host for the black fungus, and high mortality rates have been recorded in India due to the high rate of infection caused by the wide use of cow dung and urine from animals.

Medication overuse by patients has been another condition that medics have suspected that contributes to further multiplication of the disease [109].

**Figure 8:**
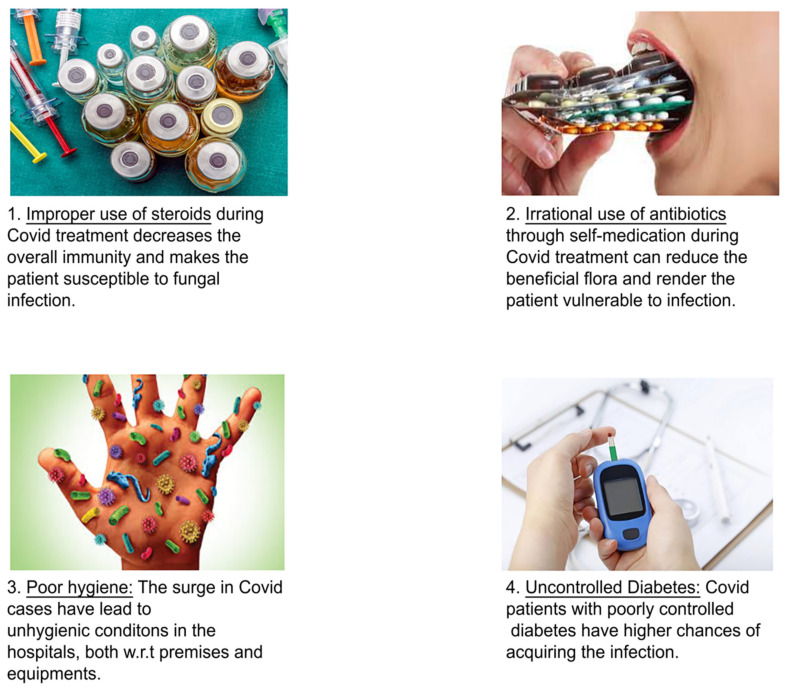
Plausible reasons for COVID patients to acquire black fungus infection.

The government has administered the rational use of antibiotics and steroids to curb the further infection rate of the disease [110]. Besides this, the government should stop the use of antibiotics and steroids over the counter to stop further infection. There is a need for early detection and quick diagnosis of the disease to stop and minimize further spread [111]. Enough testing facilities for black fungus should be made available and diabetic patients advised to control their blood sugar levels. Regular communication among the health authorities for both COVID-19 and post COVID-19 patients should be incorporated to encourage everyone to follow the health rules relating to the spread of black fungus infection. The treatment of the disease should be made affordable to minimize the rate of infection through proper treatment and medication. Figure 8 illustrates the possibility for COVID-19 patients to acquire black fungus infection.

#### 7.1.1. Diabetes and Mucormycosis: A Complex Connection

Uncontrolled diabetes is a major factor in acquiring black fungus infection in patients suffering from COVID-19 [90]. Hyperglycemia promotes fungal growth while inhibiting chemotaxis and phagocytic effectiveness. *Rhizopus oryzae*, an organism that generates the enzyme ketoreductase, which uses the ketone in the patient’s body, increases the risk of mucormycosis in diabetic ketoacidosis [45]. The blood glucose level of COVID patients increases rapidly as it affects the beta cells of pancreas. The coronavirus damages the beta cells and causes hyperglycemia.

Iron plays a vital role in the growth of mucorales, and in diabetic patients, hyperglycemia can elevate serum iron levels. In uncontrolled DM, increased blood glucose levels can glycosylate iron-sequestering proteins such as transferrin, ferritin, and lactoferrin that reduce iron affinity and cause proton-mediated displacement of ferric iron, resulting in elevation of free iron even without acidosis [112]. There can also be phagocytic dysfunction, the presence of ketone bodies like β-hydroxybutyrate [BHB], and low pH in the blood vessels that strongly impairs the ability of transferrin to chelate iron. This increased available serum iron is intracellularly transported, and enhanced expression of glucose, iron, and BHB results in the growth of fungus that augments fungal invasion followed by subsequent injury to the endothelium. Host factors suppress T lymphocyte induction, IFN-gamma production, and phagocyte-mediated killing. These factors contribute to increased lung inflammation and lung fibrosis, leading to severe disease [65]. Impaired chemotaxis and phagocytosis of neutrophils facilitate dissemination of the fungus. *Rhizopus oryzae* fungi secrete siderophores called rhizoferrin, which are low molecular weight iron chelators with more affinity than transferrin and lactoferrin. They are hydroxamate siderophores and are inefficient in obtaining iron from the serum. Hence, Mucorales that are formed in the bloodstream is readily involved in iron uptake [113]. Hemoglobin is also another source of iron that has access to heme due to its angioinvasive nature. This acts as a source of energy other than sugar which further downregulates innate immune defense mechanisms and facilitates fungal growth [114].

The Mucorales produce acute fulminant fungal rhinosinusitis and hemorrhagic necrosis at multiple sites in the brain, particularly at the base of frontal lobes and deep gray nuclei. Invaded fungi upon germination spread inferiorly to invade palate and posteriorly to invade sphenoid sinus [115]. The fungus is highly angiotropic and invades the arteries in the orbit, internal carotid artery, and cavernous sinus and produces thrombosis and hemorrhage [116]. Fungal hyphae may be seen in the perineurium, particularly the trigeminal nerve, due to the fungus’s perineural spread [117].

Mucorales use free iron by reductive iron acquisition pathway genes such as FTR1 and multicopper oxidase. The use of iron chelating agents such as deferoxamine to treat hyperferritinemia in COVID-19 infections may also predispose them to mucormycosis [118].

Mucorales spores bind to deferoxamine using their Fob1/Fob2 proteins on the cell surface, acquire iron from deferoxamine by the reductase/permease pathway, and initiate the pathological process [119]. It can also be due to the increased expression of GRP78 receptors normally seen in diabetes mellitus patients [120]. Similar to the ACE2 receptor, GRP78 also mediates endothelial cell barrier disruption and inflammation [121].

Diabetes mellitus attenuates the phagocytic functions of immune cells that allow the germination of Mucorales spores, leading to angio-invasion and tissue necrosis [122]. GRP78 and EGFR receptors are also associated with the pathogenesis of COVID-19 infections as they are essential for viral binding, internalization, and viral replication. Surprisingly, the GRP78 receptor on nasal epithelial cells and endothelial cells and integrin β1 and EGFR receptors on airway epithelial cells are essential for cell adhesion and tissue invasion by Mucorales [123]. The overexpression of the GRP78 receptor that is seen in sinus, lung, and brain tissues is associated with increased adherence and endocytosis of Rhizopus arrhizus germlings to epithelial and endothelial cells. CotH3 (spore coat protein) on the *R. arrhizus* cell surface acts explicitly as a fungal ligand for the GRP78 receptor [62]. The CotH3–GRP78 axis governs entry into the nasal epithelial and endothelial cells, explaining the increased number of ROCM (rhino-orbital cerebral mucormycosis) cases in CAM (COVID-associated mucormycosis) [63].

Increased blood sugar level stimulates fungal proliferation, due to decreased chemotaxis and phagocytic efficiency. In diabetic ketoacidosis patients, an increased risk of mucormycosis was mainly seen in *Rhizopus oryzae* invaded patients due to enzyme ketoreductase that allows the patient to utilize ketone bodies [64]. Accumulation of ketone bodies temporarily disrupts the ability of transferrin to bind to the iron. Elevation of the iron disrupts the defense mechanism and permits the growth of *Rhizopus oryzae* [65].

The mechanism in diabetic and non-diabetic patients remains the same, as in diabetic patients increased blood sugar level allows fungal spores to adhere and gives nourishment to the growth of the fungal spores. Meanwhile, in non-diabetic patients, with steroids intake, there can be increased blood sugar level, and fungal spore nourishment just needs blood sugar. Increased blood sugar can elevate the elemental free iron which can contribute to Mucorales invasion and immune suppression. Other patients who are non-diabetic or other confounding factors such as elevated zinc in bloodstream or elevated iron levels in the bloodstream, contributes to the nourishment of the Mucorales and invasion to other organs [124].

The reported mortality of mucormycosis of diabetes patients infected with COVID-19 was found to be 40–50%. With improvement in surgical debridement in the early phase of the sinonasal disease have reduced mortality to less than 10%. COVID-19 and diabetes mellitus have a bidirectional connection that has negative consequences [125]. The proinflammatory state of diabetes mellitus leads to the control of severe COVID-19 infections. Infection decreases insulin secretion through a direct pathogenic effect on pancreatic islets and forms a transient hyperinflammatory state. Subsequently, the hyperglycemia produced leads to the development of invasive mucormycosis [126].

Diabetes mellitus has an adaptive immune system, and its direct phagocytosis, suppression of neutrophil chemotaxis, and intracellular pathogen killing accelerates the infection. This leads to immune dysregulation [127]. Diabetes patients consuming corticosteroids are at high risk due to an accumulated immune suppression effect, which can increase the chance of fungal mucormycosis.

Maxillary and ethmoidal sinus mucosal thickening, as well as left-sided ptosis and proptosis with altered sensorium, were all found on facial imaging. This indicates a link between COVID-19 and diabetic ketoacidosis [128].

Endothelial adhesion and angioinvasion are important for invasive Mucorales because SARS-CoV-2 is linked to endothelial dysfunction via direct viral invasion and a host inflammatory response that causes endothelial cell death and pyroptosis. Diabetes is linked to a chronic inflammatory state that causes endothelial impairment [129].

Hyperglycemic patients who are consuming steroids have the following pathological sequences which increase the risk of morbidity and mortality to SARS-CoV-2-infected patients: induction of a phagocytic deficiency in neutrophils and macrophages; GRP78 receptor expression and upregulation in humans, as well as the Mucorales-specific protein CotH; Irin sequestering proteins are hyperglycated, causing iron sequestration to be disrupted and increased iron delivery to muscles [56].

**Figure 9:**
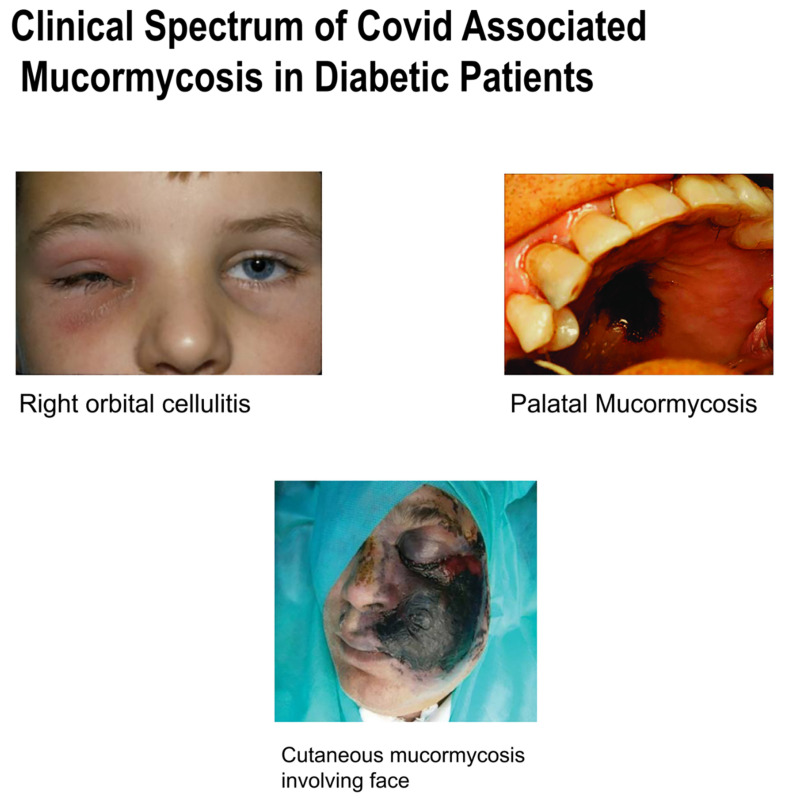
Clinical spectrum of COVID-associated mucormycosis in diabetic patients.

There is a common pathway involved in COVID-19-associated mucormycosis which is the sudden inflammatory response by the immune system. While COVID-19 is mainly associated with the entry of coronavirus through inhalation and its direct invasion onto ACE2 receptors of lungs, kidneys, heart and liver, in mucormycosis the invasion of spores can be through the nasal route, GI tract, or through injury or any other trauma. So, as such, there is no link between the two, but response to the immune system gets worsened, as it can lead to sepsis in patients having COVID-19-associated mucormycosis. The cytokine storm gets activated and pathway is paved to lymphopenia (lymphocyte count less than 1000 mm cube), followed by increased release of neutrophils and WBC. Inflammatory response gets worsened, and as a result there is increased release of IL-6 and TNF-gamma into the bloodstream. This poor inflammatory response and increased invasion of the pathogen leads to multiorgan damage and sepsis [130,131]. The clinical spectrum of COVID-associated mucormycosis in diabetic patients is explained in Figure 9.

The role of zinc supplements used in COVID-19 treatments needs to be studied for their correlation to the emergence of mucormycosis. It is also said that extensive use of steam inhalation can also cause opportunistic infection. The fungus crisis in India is not only because of improper usage of steroids among diabetic COVID patients but also over the use of medications ranging from antibiotics to zinc supplements and iron tablets.

The epithelial lining of the gastrointestinal mucosa acts as a barrier against the gut luminal content, thereby preventing the passage of elements that can cause harm to the host system. Gut-Associated Lymphoid Tissue (GALT), the well-developed and largest lymphoid immune organ in the human body, protects the host against various pathogens and infectious agents [132]. The gut bacteria, commonly called Bacteroides fragilis, promotes symbiosis and plays a vital role in intestinal immunity [133].

Overconsumption of zinc can impair this gut wall epithelium and mucosal lining of the GI tract. As a result, the lining of the GI tract can become leaky and tend to have holes. The leaky gut wall can increase proteins, gluten, microbes, and food antigen into the blood. As a result, it can breach through the tissues as well as systemic circulation, resulting in intestinal inflammation that triggers autoimmune diseases such as IBD, celiac disease, and autoimmune hepatitis [134]. Antigen-Presenting Cells (APCs), T-cells, T killer cells, B-cells, and plasma cells are immune cells activated within the intestinal barrier [135]. The leaky gut presents an inflammatory environment due to dysbiosis that paves the way to autoimmunity. Subsequent autoimmune suppression leads to microbial translocation that induces pro-inflammatory cytokines such as IFN-gamma, TNF-alpha, and IL-13. This, in turn, provides a favorable condition for the growth of mucormycetes [136]. Researchers showed that metal zinc could act as a growth factor for fungus, especially mucormycetes. It also showed that removing zinc makes it difficult for the fungus to survive. However, when the pandemic began in India, zinc supplements were used as an immunity booster.

These fungi are angioinvasive, meaning they attack the surrounding blood vessels and damage them, causing tissue necrosis and even death. The microorganism normally enters the human body through inhalation or breathing, direct inhalation from contaminated pipes and nasal prongs can also increase the risk of fungal infection. People with weakened immune systems should avoid activities like gardening and yard work involving compost piles, soil, and dust. Infection is most likely to occur in patients undergoing treatment in an ambulatory setup, in case of contaminated food, water, or any other medical instruments such as oxygen delivery tubes or prongs. ICU patients, patients with COVID-19, and cancer patients are at the highest risk of this opportunistic infection [137].

The sudden outbreak of mucormycosis is still a mystery. It is believed that the virus can damage the blood vessels and airways, and increases the susceptibility of the patients to fungal infection. COVID-19 infection can increase ferritin level, and iron increases the growth of fungus. The only consolation is that the fungus is not contagious.

#### 7.1.2. Problems Affecting the Gastrointestinal Tract during Mucormycosis Infection

Primary gastric mucormycosis is an uncommon but potentially lethal fungal disease caused by the invasion of Mucorales into the stomach mucosa. Due to an increased risk of complications in immunocompromised patients, it could result in a high fatal rate. Gastric mucormycosis is caused by long-term uncontrolled diabetes mellitus with or without diabetic ketoacidosis (DKA), solid organ or stem cell transplantation, underlying hematological malignancy, and severe trauma. Symptoms such as abdominal discomfort, hematemesis, and melena are prevalent. To establish the diagnosis, radiological imaging findings are non-specific, and a gastric biopsy is required for histological confirmation of mucormycosis. Antifungal medication is the basis of treatment, with surgical removal reserved for cases of significant disease burden or clinical deterioration [119].

Mucorales (a filamentous fungus) penetration into the stomach mucosa causes gastric mucormycosis, an uncommon but severe fungal infection that can cause substantial mortality (up to 54%) in immunocompromised patients. Mucormycosis (formally known as zygomycosis) infection is thought to be caused by the fungus class of zygomycetes, especially *Mucor*, *Rhizopus*, or *Rhizomucor* species [104].

Entry of Mucorales can be through inhalation, ingestion of contaminated food, implantation in the injured skin by trauma/burns/surgery, or by percutaneous route by contaminated needles or catheters [37]. Through the inhalation route, it can cause rhino-orbital/rhinocerebral mucormycosis or pulmonary mucormycosis; through ingestion, it can cause gastrointestinal mucormycosis; through implantation cutaneous and through inoculation, it can cause disseminated mucormycosis [86]. On immunosuppression and neutropenia, iron acts as a defense against fungal invasion due to iron elevation. This can disrupt the antimicrobial and anti-fungal properties as they secret granules that have pro-inflammatory and anti-inflammatory cytokines chemokines with fungicidal properties [138]. This is due to subsequent disruption of membrane-bound molecules that bind to endothelial cells, monocytes, and dendritic cells leading to the activation of Mucorales [93]. This leads to reduced activation of platelets and increased fungal growth. Hyphae of Mucorales also grow due to reduced NK cell secretion and direct and indirect cytotoxic effects. Other virulence factors such as secretion of lytic enzymes and release of metabolites like alkaloids or mycotoxins facilitate tissue invasion and inhibit immune response [138]. Other mechanisms such as the secretion of lipolytic or glycolytic enzymes, proteases, and subtilases also contribute to the destruction of stroma and facilitate host invasion [139].

As usual, the host defense mechanism is to damage the hyphae and phagocytose the inhaled spores and kill them. The fungal hyphae would induce chemotaxis of neutrophils that damage the hyphae [140]. These neutrophils also produce pro-inflammatory cytokines such as TNF-alpha and IFN-gamma that activate and recruit other inflammatory cells [141]. Medical care with antifungal medication, such as lipid formulations of amphotericin B, posaconazole, and newer agents isavuconazole or triazoles, is the most common treatment for stomach mucormycosis. Mucormycosis has a prevalence of 0.16 (0.12 to 0.20) per 10,000 patients [142]. Mucormycosis is common in patients with uncontrolled diabetes, with a prevalence incidence of 36%. In gastrointestinal mucormycosis, the stomach is the most afflicted organ (67%), followed by the colon (21%), small intestine (4%), and esophagus (2%). In the presence of an acidic stomach environment, phagocytic dysfunction decreased chemotaxis, and improper intracellular elimination of Mucorales are hypothesized as mechanisms of gastric mucormycosis infection. The most prevalent site of gastric mucormycosis infection is the gastric body. Blood vascular invasion beneath the mucosal surface can cause deadly gastrointestinal hemorrhage and is a poor disease predictive factor [97].

Mucormycosis is a dangerous opportunistic fungal infection that, if left untreated, can result in a high fatality rate. Early identification and therapy of disease, especially in immunocompromised patients necessitate a high index of suspicion and medical alertness. Typical presentations such as increased abdominal discomfort should be investigated using a CT scan or MRI, as well as EGD, in patients with unclear radiological imaging findings. Gastric mucormycosis is diagnosed by taking a biopsy of the probable mucosal lesions. Because of the disease’s invasive nature and high death rate, urgent medical therapy with antifungal medicines such liposomal amphotericin b or posaconazole is required. A combination of medicinal and surgical excision of mucosal lesions is indicated in individuals who have had a poor response to antifungal treatment or who have a significant disease burden.

#### 7.1.3. Pulmonary Diseases and Mucormycosis: A Complex Connection

Mucormycosis is an uncommon fungal infection that exclusively affects immunocompromised individuals and is quickly becoming a major source of infectious morbidity and mortality. Patients are usually affected by pulmonary mucormycosis by inhalation of spores. Diabetes, stem cell transplant, and hematologic malignancy are all risk factors for pulmonary mucormycosis. The first case of pulmonary mucormycosis was reported in patients with COPD. There are no specific signs and symptoms of pulmonary mucormycosis, so it is very difficult to distinguish it from other fungal infections [143].

The major treatment is to control the risk factors that cause pulmonary mucormycosis, for example, control of blood sugar level, metabolic acidosis treatment, etc. Another treatment method is the use of antifungal therapy or surgical problem of the affected tissue [28]. When compared to traditional amphotericin B deoxycholate, amphotericin B formulation is linked with reduced renal impairment [144]. The majority of azole antifungal medications are ineffective against mucormycosis. These types include voriconazole and fluconazole. Unifocal illness necessitates surgery. Bilateral disease surgery is uncommon, although it has been found to be beneficial for source control [145].

To diagnose pulmonary mucormycosis is more difficult than other fungal infections, and two effective tools are bronchoscopy, which was used in 35 of 85 patients, and percutaneous lung biopsy [146]. The diagnosis is obtained by cultures because of processing in microbiology laboratories and more aggressive bronchoscopy or surgical approaches should be pursued to obtain histopathologic specimen [38].

#### 7.1.4. Hypertension and Mucormycosis: A Complex Connection

Mucormycosis-associated mortality in hypertension and diabetes mellitus patients infected with COVID-19 are mostly of the rhino-orbital type.

Hypertensive patients with SARS-CoV-2 mostly had kidney disease or renal failure associated with mortality or morbidity risk. About 6–13% of hypertensive patients were associated with mortality [92]. Mortality was more prevalent in hypertensive patients of the older age group of >54 years of age. Renal derangement when compared with survivors had an increased odds ratio of dying to 1.75. A potentially predosing factor was found in a patient who was comorbid to hypertension with older age [93]. As in hypertensive patients, there is disruption of the RAAS mechanism followed by increased BP and vasoconstriction activity, while ACE inhibitors were commonly used for the cure of hypertension and diuretics in case of renal failure. In patients with SARS-CoV-2, as the ACE enzyme receptors become deficient due to occupation of the virus, as a result, there can be rebound hypertension, which is followed by a significant decrease in the immune system that contributes to humongous invasion of Mucorales [6].

## 8. Prospects and Recommendations

As the cases of black fungus rise rapidly, it forces the government toward new medications and treatment protocols. Early detection and aggressive treatment are key to survival in COVID-Associated Mucormycosis (CAM) [90]. One such recommendation other than amphotericin B was brought by the Bajaj healthcare team to release posaconazole (injectable), a triazole, an anti-fungal agent that considerably eases the pressure and offers patients much needed and timely therapy options. Oral tablets of posaconazole are preferred as step-down treatment and continued for three to six months to prevent a recurrence [147].

As mortality is expected more in patients with uncontrolled diabetes mellitus, administering the correct dose and duration of steroids can halt such infections [110]. According to the ‘Illness to Wellness’ campaign with the theme Diabetes Care and Management During the Post COVID–19 Era, improved multi-disciplinary management can reduce the prevalence of black fungus infections. All individuals must preach and practice healthy habits for a good life, including intake of a balanced diet, regular exercise, and adequate and proper sleep that are important to avoid stress [37].

## 9. Conclusions

Though the etiopathogenesis of mucormycosis illness differs by nation, the disease’s appearance may be somewhat severe, with a high death rate if not treated quickly. As a result, many doctors find it challenging to deal with it. Given the high mortality rate, early and quick diagnosis and an effort to recover from the predisposing circumstances are critical to properly treating this illness. Early intervention, such as surgical debridement and medicines, can help to improve the status of this dangerous illness.

## Figures and Tables

**Table 1:** An overview on the different types of nanotechnology based diagnostic methods.

Test	Uses	Advantages	Disadvantages
Fungal detection biosensors	For diagnosis of invasive aspergillosis [62] Detection of mycotoxins Detection of fungal pathogens in agriculture [63]	High specificity Very less usage of reagents required for calibration Fast response time [64]	Poor sensitivity for many of the clinically relevant targets [65]
Diagnostics based on nucleic acids	Detection of fungal infections Diagnosis of cancer Identification of genetic markers related to cancer	Able to detect genetic markers in case of infectious diseases Identify organisms without long isolation processes	Expensive Radioactivity needed False positives can occur [64].
Point of Care tests	Detecting presence of fungal pathogens Blood glucose monitoring Temperature monitoring Rapid strep tests	Efficiency Speedy diagnosis Expanded testing capabilities [64]	Experienced and trained staff is required Lack of cost-effectiveness [65]
Detection of Galactomannans	Diagnosis of invasive aspergillosis in conditions like acute leukemia and hematopoietic stem cell transplantation	Early diagnosis Used for follow-up of therapy Can detect even small levels	False positive or negative results can occur

## References

[1] Wuhan City Health Committee Wuhan Municipal Health and Health Commission’s Briefing on the Current Pneumonia Epidemic Situation in Our City 2019. http://wjw.wuhan.gov.cn/front/web/showDetail/2019123108989.

[2] Frazier K.M., Hooper J.E., Mostafa H.H., Stewart C.M. (2020). SARS-CoV-2 virus isolated from the mastoid and middle ear: Implications for COVID-19 precautions during ear surgery: Implications for COVID-19 precautions during ear surgery. JAMA Otolaryngol. Head Neck Surg..

[3] Ferguson B.J. (2000). Definitions of fungal rhinosinusitis. Otolaryngol. Clin. N. Am..

[4] Chakrabarti A., Denning D.W., Ferguson B.J., Ponikau J., Buzina W., Kita H., Marple B., Panda N., Vlaminck S., Kauffmann-Lacroix C. (2009). Fungal rhinosinusitis: A categorization and definitional schema addressing current controversies: A categorization and definitional schema addressing current controversies. Laryngoscope.

[5] Mohindra S., Gupta R., Bakshi J., Gupta S.K. (2007). Rhinocerebral mucormycosis: The disease spectrum in 27 patients. Mycoses.

[6] Munir N., Jones N.S. (2007). Rhinocerebral mucormycosis with orbital and intracranial extension: A case report and review of optimum management. J. Laryngol. Otol..

[7] DeShazo R.D., Chapin K., Swain R.E. (1997). Fungal sinusitis. N. Engl. J. Med..

[8] Gillespie M.B., O’Malley B.W. (2000). An algorithmic approach to the diagnosis and management of invasive fungal rhinosinusitis in the immunocompromised patient. Otolaryngol. Clin. N. Am..

[9] González Ballester D., González-García R., Moreno García C., Ruiz-Laza L., Monje Gil F. (2012). Mucormycosis of the head and neck: Report of five cases with different presentations. J. Craniomaxillofac. Surg..

[10] Chen N., Zhou M., Dong X., Qu J., Gong F., Han Y., Qiu Y., Wang J., Liu Y., Wei Y. (2020). Epidemiological and clinical characteristics of 99 cases of 2019 novel coronavirus pneumonia in wuhan, china: A descriptive study. Lancet.

[11] Song G., Liang G., Liu W. (2020). Fungal co-infections associated with global covid-19 pandemic: A clinical and diagnostic perspective from china. Mycopathologia.

[12] Yang X., Yu Y., Xu J., Shu H., Xia J., Liu H., Wu Y., Zhang L., Yu Z., Fang M. (2020). Clinical course and outcomes of critically ill patients with sars-cov-2 pneumonia in wuhan, china: A single-centered, retrospective, observational study. Lancet Respir. Med..

[13] Gangneux J.P., Bougnoux M.E., Dannaoui E., Cornet M., Zahar J.R. (2020). Invasive fungal diseases during covid-19: We should be prepared. J. Mycol. Med..

[14] ‘Black Fungus’ Disease Linked to Covid Spreads across India. The Guardian. https://www.theguardian.com/world/2021/may/21/mucormycosis-black-fungus-disease-linked-covid-spreads-india.

[15] India Sees 259,551 New Covid Cases, ‘Black Fungus’ Adds to Woes. Aljazeera. https://www.google.com/amp/s/www.aljazeera.com/amp/news/2021/5/21/india-sees-259551-new-covid-cases-black-fungus-adds-to-woes.

[16] Mucormycosis: Can Black Fungus Infect People Who Don’t Have Covid-19? Here’s What Experts Say. Money Control News. https://www.moneycontrol.com/news/india/mucormycosis-can-black-fungus-infect-people-without-covid-19-heres-what-experts-say-6929921.html.

[17] COVID-19 and Black Fungus: What Is Mucormycosis? Health, the Sciences. https://science.thewire.in/the-sciences/covid-19-and-black-fungus-what-is-mucormycosis/.

[18] Mucormycosis in COVID-19 Patients: Everything You Should Know. Hetero Health Care. https://www.heterohealthcare.com/blog/what-is-the-fungal-infection-mucormycosis-affecting-covid-patients-in-india.

[19] Mucormycosis/Black Fungus: Why Are COVID-19 Patients at Risk?. https://www.netmeds.com/health-library/post/mucormycosis-black-fungus-why-are-covid-19-patients-at-risk-here-are-icmr-guidelines-for-prevention.

[20] Mucormycosis. CDC. https://www.cdc.gov/fungal/diseases/mucormycosis/index.html.

[21] Mucormycosis; the Black Fungus Hitting Covid Patients. BBC. https://www.bbc.com/future/article/20210519-mucormycosis-the-black-fungus-hitting-indias-covid-patients.

[22] Black fungus or Mucormycosis: Costly Mistakes in COVID-19 Treatment Lead to New Challenges. First Point. https://www.firstpost.com/india/black-fungus-or-mucormycosis-costly-mistakes-in-covid-19-treatment-lead-to-new-challenges-9637481.html.

[23] Mucormycosis: An Emerging Challenge in COVID-19 Management. https://health.economictimes.indiatimes.com/news/industry/mucormycosis-an-emerging-challenge-in-covid-19-management/82507750.

[24] Katragkou A., Walsh T.J., Roilides E. (2014). Why is mucormycosis more difficult to cure than more common mycoses?. Clin. Microbiol. Infect. Off. Publ. Eur. Soc. Clin. Microbiol. Infect. Dis..

[25] Mehta S., Pandey A. (2020). Rhino-orbital mucormycosis associated with COVID-19. Cureus.

[26] Prakash H., Chakrabarti A. (2019). Global epidemiology of mucormycosis. J. Fungi.

[27] Prakash H., Ghosh A.K., Rudramurthy S.M., Singh P., Xess I., Savio J., Pamidimukkala U., Jillwin J., Varma S., Das A. (2019). A prospective multicenter study on mucormycosis in india: Epidemiology, diagnosis, and treatment. Med. Mycol..

[28] Kontoyiannis D.P., Yang H., Song J., Kelkar S.S., Yang X., Azie N., Harrington R., Fan A., Lee E., Spalding J.R. (2016). Prevalence, clinical and economic burden of mucormycosis-related hospitalizations in the united states: A retrospective study. BMC Infect. Dis..

[29] Guinea J., Escribano P., Vena A., Muñoz P., Martínez-Jiménez M.D.C., Padilla B., Bouza E. (2017). Increasing incidence of mucormycosis in a large spanish hospital from 2007 to 2015: Epidemiology and microbiological characterization of the isolates. PLoS ONE.

[30] Ambrosioni J., Bouchuiguir-Wafa K., Garbino J. (2010). Emerging invasive zygomycosis in a tertiary care center: Epidemiology and associated risk factors. Int. J. Infect. Dis..

[31] Muthu V., Rudramurthy S.M., Chakrabarti A., Agarwal R. (2021). Epidemiology and pathophysiology of covid-19-associated mucormycosis: India versus the rest of the world. Mycopathologia.

[32] 760 Black Fungus Patients Still Being Treated in Delhi Hospitals. The Hindustan Times. https://www.hindustantimes.com/cities/delhi-news/760-black-fungus-patients-still-being-treated-in-delhi-hospitals-101625248850429.html.

[33] PTI Over 3000 Affected by Black Fungus, 122 Fatalities in TN: Minister. The Indian Express. https://indianexpress.com/article/cities/chennai/over-3000-affected-black-fungus-122-fatalities-tn-minister-7392597/lite/.

[34] Black Fungus: ICMR Releases Guidelines: Dos and Don’ts. India TV. https://www.indiatvnews.com/fyi/mucormycosis-epidemic-icmr-releases-guidelines-dos-and-donts-for-black-fungus-infection-symptoms-706050.

[35] ICMR Releases Diagnosis and Management Guidelines for COVID-19 Associated Mucormycosis. Firstpost. https://www.google.com/amp/s/www.firstpost.com/india/icmr-releases-diagnosis-and-management-guidelines-for-covid-19-associated-mucormycosis-9628341.html/amp.

[36] Mohammadi R., Nazeri M., Sayedayn S.M., Ehteram H. (2014). A successful treatment of rhinocerebralmucormycosis due to Rhizopusoryzae. J. Res. Med. Sci..

[37] Spellberg B., Edwards J., Ibrahim A. (2005). Novel perspectives on mucormycosis: Pathophysiology, presentation, and management. Clin. Microbiol. Rev..

[38] Roden M.M., Zaoutis T.E., Buchanan W.L., Knudsen T.A., Sarkisova T.A., Schaufele R.L., Walsh T.J. (2005). Epidemiology and outcome of zygomycosis: A review of 929 reported cases. Clin. Infect. Dis. Off. Publ. Infect. Dis. Soc. Am..

[39] Mucormycosis: The ‘Black Fungus’ Maiming Covid Patients in India. BBC. https://www.bbc.com/news/world-asia-india-57027829.

[40] India Today Web Desk Andhra Pradesh Sees Rise in Black Fungus Cases, Active Case Tally at 677. https://www.indiatoday.in/india/story/andhra-pradesh-sees-rise-in-black-fungus-cases-1838920-2021-08-10.

[41] Guegan H., Iriart X., Bougnoux M.E., Berry A., Robert-Gangneux F., Gangneux J.P. (2020). Evaluation of MucorGenius® mucorales PCR assay for the diagnosis of pulmonary mucormycosis. J. Infect..

[42] Sen M., Lahane S., Lahane T.P., Parekh R., Honavar S.G. (2021). Mucor in a viral land: A tale of two pathogens. Indian J. Ophthalmol..

[43] Prattes J., Valentine T., Hoengil M., Talakic E., Reisinger A., Philipp E. (2021). Invasive pulmonary aspergillosis complicating COVID-19 in the ICU—A case report. Med. Mycol. Case Rep..

[44] Hage C.A., Carmona E.M., Epelbaum O., Evans S.E., Gabe L.M., Haydour Q., Limper A.H. (2009). Microbiological laboratory testing in the diagnosis of fungal infections in pulmonary and critical care practice. An official American thoracic society clinical practice guideline. Am. J. Respir. Crit. Care Med..

[45] Cornely O.A., Alastruey-Izquierdo A., Arenz D., Chen S.C.A., Dannaoui E., Hochhegger B., Chakrabarti A. (2019). Global guideline for the diagnosis and management of mucormycosis: An initiative of the European confederation of medical mycology in cooperation with the mycoses study group education and research consortium. Lancet Infect. Dis..

[46] Skiada A., Lass-Floerl C., Klimko N., Ibrahim A., Roilides E., Petrikkos G. (2018). Challenges in the diagnosis and treatment of mucormycosis. Med Mycol..

[47] Bellanger A.P., Berceanu A., Rocchi S., Valot B., Fontan J., Chauchet A., Millon L. (2018). Development of a quantitative PCR detecting Cunninghamella bertholletiae to help in diagnosing this rare and aggressive mucormycosis. Bone Marrow Transplant..

[48] Brunet K., Rammaert B. (2020). Mucormycosis treatment: Recommendations, latest advances, and perspectives. J. Mycol. Méd..

[49] Pakdel F., Ahmadikia K., Salehi M., Tabari A., Jafari R., Mehrparvar G., Khodavaisy S. (2020). Mucormycosis in patients with COVID-19: A cross-sectional descriptive multicentre study from Iran. Mycoses.

[50] Santana O.I., Silva J.A.G., Andrade C.A.S., Lima O.M.D. (2019). Biosensors for early detection of fungi spoilage and toxigenic and mycotoxins in food. Curr. Opin. Food Sci..

[51] Hussain K., Malavia D., Johnson E.M., Littlechild J., Winlove C.P., Vollmer F., Gow N.A.R. (2020). Biosensors and diagnostics for fungal detection. J. Fungi..

[52] Biosensor Technology: Advantages and Applications. https://www.azosensors.com/article.aspx?ArticleID=402.

[53] Morales-Del Ángel J.A., Morales-Chávez O.D., Méndez-Sáenz M.A., Montemayor-Alatorre A., Jasso-Ramírez N.G., Treviño-González J.L. (2020). Clinical Implications of facial edema in chronic mucormycosis: A report of 5 cases. Arch. Clin. Med. Case Rep..

[54] Hoffmann C., Guillerm G., Le Pape P., Carausu L., Lavergne R.A., Nevez G., Le Gal S. (2020). Mucorales DNA detection in serum specimens for early diagnosis of mucormycosis. Diagn. Microbiol. Infect. Dis..

[55] Liu M., Bruni G.O., Taylor C.M., Zhang Z., Wang P. (2018). Comparative genome-wide analysis of extracellular small RNAs from the mucormycosis pathogen Rhizopus delemar. Sci. Rep..

[56] Varga Z., Flammer A.J., Steiger P., Haberecker M., Andermatt R., Zinkernagel A.S. (2020). Endothelial cell infection and endotheliitis in COVID-19. Lancet.

[57] Haidar G., Singh N. (2018). How we approach combination antifungal therapy for invasive aspergillosis and mucormycosis in transplant recipients. Transplantation.

[58] Khan Z., Ahmad S. (2020). Diagnostic Algorithm for Invasive Fungal Infections: In Clinical Practice of Medical Mycology in Asia.

[59] Jafari M., Abolmaali S.S., Tamaddon A.M., Zomorodian K., Shahriarirad B. (2021). Nanotechnology approaches for delivery and targeting of Amphotericin B in fungal and parasitic diseases. Nanomedicine.

[60] Zhou W., Li H., Zhang Y., Huang M., He Q., Li P., Zhang F., Shi Y., Su X. (2017). Diagnostic value of galactomannan antigen test in serum and bronchoalveolar lavage fluid samples from patients with nonneutropenic invasive pulmonary aspergillosis. J. Clin. Microbiol..

[61] Baby B., Devan A.R., Nair B., Nath L.R. (2020). The impetus of COVID-19 in multiple organ affliction apart from respiratory infection: Pathogenesis, diagnostic measures and current treatment strategy. Infect. Disord. Drug Targets.

[62] Gleissner B., Schilling A., Anagnostopolous I., Siehl I., Thiel E. (2004). Improved outcome of zygomycosis in patients with hematological diseases?. Leuk. Lymphoma.

[63] Timpl R., Rohde H., Robey P.G., Rennard S.I., Foidart J.M., Martin G.R. (1979). Laminin—A glycoprotein from basement membranes. J. Biol. Chem..

[64] Bouchara J.P., Oumeziane N.A., Lissitzky J.C., Larcher G., Tronchin G., Chabasse D. (1996). Attachment of spores of the human pathogenic fungus Rhizopus oryzae to extracellular matrix components. Eur. J. Cell Biol..

[65] Liu M., Spellberg B., Phan Q.T., Fu Y., Lee A.S., Edwards J.E. (2010). The endothelial cell receptor GRP78 is required for mucormycosis pathogenesis in diabetic mice. J. Clin. Investig..

[66] Dadwal S.S., Kontoyiannis D.P. (2018). Recent advances in the molecular diagnosis of mucormycosis. Expert Rev. Mol. Diagn..

[67] Herbrecht R., Denning T.F., Patterson J.E., Bennett R.E., Greene J.W., Oestmann W., Kern V. (2002). Voriconazole versus amphotericin B for primary therapy of invasive aspergillosis. N. Engl. J. Med..

[68] Stone E.A., Fung H.B., Kirschenbaum H.L. (2002). Caspofungin: An echinocandin antifungal agent. Clin. Ther..

[69] Silva L.N., de Mello T.P., de Souza Ramos L. (2019). Current challenges and updates on the therapy of fungal infections. Curr. Top. Med. Chem..

[70] Chowdhary A., Sharma C., Meis J.F. (2017). Azole resistant aspergillosis: Epidemiology, molecular mechanisms and treatment. J. Infect. Dis..

[71] Perlin D.S. (2015). Echinocandin resistance in Candida. Clin. Intect. Dis..

[72] Cai S., Sun W., Li M., Dong L.A. (2020). Complex COVID 19 case with rheumatoid arthritis treated with toclizumab. Clin. Rheumatol..

[73] Sahoo J.P., Mishra A.P., Pradhan P., Samal K.C. (2021). Misfortune never comes alone—The new “black fungus” accompanying COVID-19 wave. Biot. Res. Today.

[74] Sugar A.M. (1995). Use of amphotericin B with azole antifungal drugs: What are we doing?. Antimicrob. Agents Chemother..

[75] Adler-Moore J., Richard T.P. (2002). AmBisome: Liposomal formulation, structure, mechanism of action and pre-clinical experience. J. Antimicrob. Chemother..

[76] De Beule K., Van Gestel J. (2001). Pharmacology of itraconazole. Drugs.

[77] Hof H. (2006). A new, broad-spectrum azole antifungal: Posaconazole--mechanisms of action and resistance, spectrum of activity. Mycoses.

[78] Brattsand R., Linden M. (1996). Cytokine modulation by glucocorticoids: Mechanisms and actions in cellular studies. Aliment. Pharm. Ther..

[79] Spellberg B., Walsh T.J., Kontoyiannis D.P. (2009). Recent advances in the management of mucormycosis: From bench to bedside. Clin. Infect. Dis..

[80] Lin E., Moua T., Limper A.H. (2017). Pulmonary mucormycosis: Clinical features and outcomes. Infection.

[81] Lalayanni C., Baliakas P., Xochelli A., Apostolou C., Arabatzis M., Velegraki A. (2012). Outbreak of cutaneous zygomycosis associated with the use of adhesive tape in haematology patients. J. Hosp. Infect..

[82] Szalai G., Fellegi V., Szabó Z., Vitéz L.C. (2006). Mucormycosis mimics sinusitis in a diabetic adult. Ann. N. Y. Acad. Sci..

[83] Losee J.E., Selber J., Vega S., Hall C., Scott G., Serletti J.M. (2002). Primary cutaneous mucormycosis: Guide to surgical management. Ann. Plast. Surg..

[84] Chew H.H., Abuzeid A., Singh D. (2008). Surgical wound mucormycosis necessitating hand amputation: A case report. J. Orthop. Surg..

[85] Ingram P.R., Suthananthan A.E., Rajan R., Sieunarine K., Gardam D.J. (2014). Cutaneous mucormycosis and motor vehicle accidents: Findings from an Australian case series. Med. Mycol..

[86] Sun H.Y., Singh N. (2011). Mucormycosis: Its contemporary face and management strategies. Lancet Infect. Dis..

[87] Polo J.R., Luño J., Menarguez C., Gallego E., Robles R. (2009). Peritoneal mucormycosis in a patient receiving continuous ambulatory peritoneal dialysis. Am. J. Kidney Dis..

[88] Mucormycosis (Zygomycosis) Treatment & Management. https://emedicine.medscape.com/article/222551-treatment.

[89] Torres-Narbona M., Guinea A., Muñoz P., Bouza E. (2007). Zigomicetos y zigomicosis en la era de las nuevas terapias antifúngicas [Zygomycetes and zygomycosis in the new era of antifungal therapies]. Rev. Esp. Quim..

[90] Goel S., Palaskar S., Shetty V.P., Bhushan A. (2009). Rhinomaxillary mucormycosis with cerebral extension. J. Oral Maxillofac. Pathol..

[91] Ali T., Kaitha S., Mohmood S., Ftesi A., Stone J., Bronze M.S. (2013). Clinical use of anti TNF therapy and increased risk of infection. Drug. Health Patient Saf..

[92] Hadi H.A., Suwaidi J.A. (2020). Endothelial dysfunction in diabetes mellitus. Vasc. Health Risk. Manag..

[93] Ibrahim A.S., Spellberg B., Walsh T.J., Kontoyiannis D.P. (2021). Pathogenesis of mucormycosis. Clin. Infect. Dis. Off. Publ. Infect. Dis. Soc. Am..

[94] Werthman-Ehrenreich A. (2021). Mucormycosis with orbital compartment syndrome in a patient with COVID-19. Am. J. Emerg. Med..

[95] Ravani S.A., Agrawal G.A., Leuva P.A., Modi P.H., Amin K.D. (2021). Rise of the phoenix: Mucormycosis in COVID-19 times. Indian J. Ophthalmol..

[96] Patel A., Agarwal R., Rudramurthy S.M., Shevkani M., Xess I., Sharma R. (2021). Multicenter epidemiologic study of Coronavirus disease-associated mucormycosis, India. Emerg. Infect. Dis..

[97] Ibrahim A.S., Kontoyiannis D.P. (2013). Update on mucormycosis pathogenesis. Curr. Opin. Infect. Dis..

[98] Bhadra A., Ahmed M.S., Rahman M.A., Islam S. (2021). Mucormycosis or black fungus: An emerging threat in COVID-19. Bangabandhu Sheikh Mujib Med. Univ. J..

[99] Daria S., Asaduzzaman M., Shahriar M., Islam M.R. (2021). The massive attack of COVID-19 in India is a big concern for Bangladesh: The key focus should be given on the interconnection between the countries. Int. J. Health Plan. Manag..

[100] Ezeokoli O.T., Gcilitshana O., Pohl C.H. (2021). Risk factors for fungal co-infections in critically ill COVID-19 patients, with a focus on immunosuppressants. J. Fungi..

[101] Daria S., Islam M.R. The use of cow dung and urine to cure COVID-19 in India: A public health concern. Int. J. Health Plan. Manag..

[102] Sankar S., Gokhale T., Choudhury S.S., Deb A.K. (2021). COVID 19 and orbital mucormycosis. Indian J. Ophthalmol..

[103] Berenner E.J., Ungaro R.C., Gearry R.B., Zhang X., Colombel J.F., Kappelman M.D. (2020). Corticosteroids, but not TNF antagonists, are associated with adverse COVID 19 outcomes in patient with inflammatory bowel diseases. Gastroenterology.

[104] Artis W.M., Fountain J.A., Delcher H.K., Jones H.E. (1982). A mechanism in susceptibility to mucormycosids in diabetic ketoacidosis: Transferrin and iron availability. Diabetes.

[105] Pagano L., Ricci P., Tonso A., Nosari A.M., Cudillo L., Montillo M., Cenacchi A. (1999). Mucormycosis in patients with haematological malignancies: A retrospective clinical study of 37 cases. Br. J. Haematol..

[106] Sharma S.R., Sharma B. (2021). Opportunistic fungal infections post-COVID: How threatened are we?. Homœopath. Links.

[107] Mahalaxmi I., Jayaramayya K., Venkatesan D., Subramaniam M.D., Renu K., Vijayakumar P., Vellingiri B. (2021). Mucormycosis: An opportunistic pathogen during COVID-19. Environ. Res..

[108] Forouzesh M., Farshid S., Ghiasi N., Narimani M., Valizadeh R., Sadighpour T., Alimohammadi S. (2021). Mucormycosis (black fungus/zygomycosis) and COVID-19; Does the coexistence of these two increase mortality?. Immunopathol. Persa.

[109] Gandra S., Ram S., Levitz S.M. (2021). The “black fungus” in India: The emerging syndemic of COVID-19—Associated mucormycosis. Antibiotics.

[110] Garg E., PulinSaluja A.D., Khurana C., Arora M., Rai R. (2021). Mucormycosis: The black fungus–An insidious killer. Ann. Rom. Soc. Cell Biol..

[111] Shevade D.S. (2021). Mucormycosis: Black fungus, a deadly post-COVID infection. Microbiology.

[112] Ibrahim A.S. (2011). Host cell invasion in mucormycosis: Role of iron. Curr. Opin. Microbiol..

[113] Challa S., Uppin S.G., Hanumanthu S., Panigrahi M.K., Purohit A.K., Sattaluri S. (2010). Fungal rhinosinusitis: A clinicopathological study from South India. Eur. Arch. Otorhinolaryngol..

[114] Sundaram C., Mahadevan A., Laxmi V., Yasha T.C., Santosh V., Murthy J.M. (2005). Cerebral zygomycosis. Mycoses.

[115] Neblett F.R., Benedict K., Bos J., Bennett S.D., Lo Y.C., Adebanjo T. (2012). Necrotizing cutaneous mucormycosis after a tornado in Joplin, Missouri, in 2011. N. Engl. J. Med..

[116] Andresen D., Donaldson A., Choo L., Knox A., Klaassen M., Ursic C. (2005). Multifocal cutaneous mucormycosis complicating polymicrobial wound infections in a tsunami survivor from Sri Lanka. Lancet.

[117] Alqarihi A., Gebremariam T., Gu Y., Swidergall M., Alkhazraji S., Soliman S.S.M., Bruno V.M., Edwards J.E., Filler S.G., Uppuluri P. (2020). GRP78 and integrins play different roles in host cell invasion during Mucormycosis. Mbio.

[118] Gebremariam T., Liu M., Luo G., Bruno V., Phan Q.T., Waring A.J., Edwards J.E., Filler S.G., Yeaman M.R., Ibrahim A.S. (2014). CotH3 mediates fungal invasion of host cells during mucormycosis. J. Clin. Investig..

[119] Marx R.E., Stern D., Marx R.E., Stern D. (2009). Inflammatory, Reactive and Infectious Diseases. Oral and Maxillofacial Pathology.

[120] CAsqueiro J., Casqueiro J., Alves C. (2012). Infections in Patients with Diabetes Mellitus: A Review of Pathogenesis. Indian J. Endocrinol. Metab..

[121] Ibrahim A.S., Edwards J.E.J., Filler S.G., Dismukes W.E., Pappas P.G., Sobel J.D. (2003). Zygomycosis. Clinical Mycology.

[122] Eucker J., Sezer O., Graf B., Possinger K. (2001). Mucormycoses. Mycoses.

[123] Tribble D.R., Rodriguez C.J. (2014). Combat-related invasive fungal wound infections. Curr. Fungal Infect. Rep..

[124] Nair A.G., Adulkar N.G., D’Cunha L., Rao P.R., Bradoo R.A., Bapaye M.M., Kothari A., Dave T.V., Shinde C.A. (2021). Rhino-orbital Mucor mycosis following COVID-19 in previously non-diabetic, immunocompetent patients. Orbit.

[125] Kontoyiannis D.P., Lewis R.E. (2015). Agents of mucormycosis and entomophthoramycosis. Mandell Douglas Bennett’s Princ. Pract. Infect. Dis..

[126] Jung J., Kim M.Y., Lee H.J., Park Y.S., Lee S.O., Choi S.H., Kim S.H. (2015). Comparison of computed tomographic findings in pulmonary mucormycosis and invasive pulmonary aspergillosis. Clin. Microbiol. Infect. Off. Publ. Eur. Soc. Clin. Microbiol. Infect. Dis..

[127] Legrand M., Gits-Muselli M., Boutin L., Garcia-Hermoso D., Maurel V., Soussi S., Alanio A. (2016). Detection of circulating Mucorales DNA in critically ill burn patients: Preliminary report of a screening strategy for early diagnosis and treatment. Clin. Infect. Dis. Off. Publ. Infect. Dis. Soc. Am..

[128] Hussain A., Bhowmik B., do Vale Moreira N.C. (2020). COVID-19 and diabetes: Knowledge in progress. Diabetes Res. Clin. Pract..

[129] Ceriello A., De Nigris V., Prattichizzo F. (2020). Why is hyperglycemia worsening COVID-19 and its prognosis?. Diabetes Obes. Metab..

[130] Farah Y., Hala N., Aisha N., Dapk K.E., Phadke R. (2021). COVID-19 associated mucor mycosis: A systematic review from diagnostic challenges to management. Disease.

[131] Tay M.Z., Poh C.M., Rénia L., Macary P.A., Ng L.F.P. (2020). The trinity of COVID-19: Immunity, inflammation and intervention. Nat. Rev. Immunol..

[132] Kumar M., Kumar S.D., Shubham S., Kumawat M., Verma V., Singh B. (2021). Mucormycosis in COVID-19 pandemic: Risk factors and linkages. Clin. Res. Microb. Sci..

[133] Fasano A., Baudry B., Pumplin D.W., Wasserman S.S., Tall B.D., Ketley J.M., Kaper J.B. (1991). Vibrio cholerae produces a second enterotoxin, which affects intestinal tight junctions. Proc. Natl. Acad. Sci. USA.

[134] Fasano A., Shea-Donohue T. (2005). Mechanisms of disease: The role of intestinal barrier function in the pathogenesis of gastrointestinal autoimmune diseases. Nat. Clin. Pract. Gastroenterol. Hepatol..

[135] Abdelhamid L., Luo X.M. (2018). Retinoic acid, leaky gut, and autoimmune diseases. Nutrients.

[136] Nikitakis N.G., Papaioannou W., Sakkas L.I., Kousvelari E. (2017). The autoimmunity-oral microbiome connection. Oral Dis..

[137] Garg D., Muthu V., Sehgal I.S., Ramachandran R., Kaur H., Bhalla A. (2021). coronavirus disease (Covid-19) associated mucormycosis (CAM): Case report and systematic review of literature. Mycopathologia.

[138] Hood M.I., Skaar E.P. (2012). Nutritional immunity: Transition metals at the pathogen-host interface. Nat. Rev. Microbiol..

[139] Roilides E., Kontoyiannis D.P., Walsh T.J. (2012). Host defenses against zygomycetes. Clin. Infect. Dis..

[140] Chamilos G., Lewis R.E., Lamaris G., Walsh T.J., Kontoyiannis D.P. (2008). Zygomycetes hyphae trigger an early, robust proinflammatory response in human polymorphonuclear neutrophils through toll-like receptor 2 induction but display relative resistance to oxidativedamage. Antimicrob. Agents Chemother..

[141] Binder U., Maurer E., Lass-Flörl C. (2014). Mucormycosis—From the pathogens to the disease. Clin. Microbiol. Infect..

[142] Death of Women with Black Fungus Symptom Causes a Scare. The Hindu. https://www.thehindu.com/news/national/andhra-pradesh/death-of-woman-with-black-fungus-symptoms-causes-a-scare/article34600320.ece.

[143] Uchida T., Okamoto M., Fujikawa K., Yoshikawa D., Mizokami A., Mihara T., Kawakami A. (2019). Gastric mucormycosis complicated by a gastropleural fistula: A case report and review of the literature. Medicine.

[144] Dioverti M.V., Cawcutt K.A., Abidi M., Sohail M.R., Walker R.C., Osmon D.R. (2015). Gastrointestinal mucormycosis in immunocompromised hosts. Mycoses.

[145] Agarwal R., Kumar V., Gupta D. (2006). Pulmonary mucormycosis: Two of a kind. Eur. J. Intern. Med..

[146] Panigrahi M.K., Manju R., Kumar S.V., Toi P.C. (2014). Pulmonary mucormycosis presenting as nonresolving pneumonia in a patient with diabetes mellitus. Respir. Care.

[147] Raza A.F., Paudel D.R., Prabhu P. (2021). Black fungus and COVID-19: Role of otorhinolaryngologists and audiologists. Eur. Arch. Oto-Rhino-Laryngol..

